# In Vitro and Molecular Docking Evaluation of the Anticholinesterase and Antidiabetic Effects of Compounds from *Terminalia macroptera* Guill. & Perr. (Combretaceae)

**DOI:** 10.3390/molecules29112456

**Published:** 2024-05-23

**Authors:** Romeo Toko Feunaing, Alfred Ngenge Tamfu, Abel Joel Yaya Gbaweng, Selcuk Kucukaydin, Joseph Tchamgoue, Alain Meli Lannang, Bruno Ndjakou Lenta, Simeon Fogue Kouam, Mehmet Emin Duru, El Hassane Anouar, Emmanuel Talla, Rodica Mihaela Dinica

**Affiliations:** 1 Department of Chemistry, Faculty of Sciences, University of Ngaoundere, Ngaoundere P.O. Box 454, Cameroon; 2 Department of Chemical Engineering, School of Chemical Engineering and Mineral Industries, University of Ngaoundere, Ngaoundere P.O. Box 454, Cameroon; 3 Department of Medical Services and Techniques, Koycegiz Vocational School of Health Services, Mugla Sitki Kocman University, 48800 Mugla, Turkey; 4 Department of Chemistry, Faculty of Science, Mugla Sitki Kocman University, 48000 Mugla, Turkey; 5 Department of Chemistry, Physics and Environment, Faculty of Sciences and Environment, ‘Dunarea de Jos University’, 47 Domneasca Str., 800008 Galati, Romania; 6 Department of Chemistry, Higher Teacher Training College, The University of Yaoundé 1, Yaoundé P.O. Box 47, Cameroon; 7 Department of Organic Chemistry, Faculty of Science, University of Yaounde 1, Yaoundé P.O. Box 812, Cameroon; 8 Department of Chemistry, College of Sciences and Humanities in Al-Kharj, Prince Sattam bin Ab-dulaziz University, Al-Kharj P.O. Box 83, Saudi Arabia

**Keywords:** *Terminalia macroptera*, diabetes, Alzheimer’s disease, cholinesterase inhibition, α-glucosidase inhibition, α-amylase inhibition, molecular docking

## Abstract

Alzheimer’s disease (AD) and diabetes are non-communicable diseases with global impacts. Inhibitors of acetylcholinesterase (AChE) and butyrylcholinesterase (BChE) are suitable therapies for AD, while α-amylase and α-glucosidase inhibitors are employed as antidiabetic agents. Compounds were isolated from the medicinal plant *Terminalia macroptera* and evaluated for their AChE, BChE, α-amylase, and α-glucosidase inhibitions. From ^1^H and ^13^C NMR data, the compounds were identified as 3,3′-di-O-methyl ellagic acid (**1**), 3,3′,4′-tri-O-methyl ellagic acid-4-O-β-D-xylopyranoside (**2**), 3,3′,4′-tri-O-methyl ellagic acid-4-O-β-D-glucopyranoside (**3**), 3,3′-di-O-methyl ellagic acid-4-O-β-D-glucopyranoside (**4**), myricetin-3-O-rhamnoside (**5**), shikimic acid (**6**), arjungenin (**7**), terminolic acid (**8**), 24-deoxysericoside (**9**), arjunglucoside I (**10**), and chebuloside II (**11**). The derivatives of ellagic acid (**1**–**4**) showed moderate to good inhibition of cholinesterases, with the most potent being 3,3′-di-O-methyl ellagic acid, with IC_50_ values of 46.77 ± 0.90 µg/mL and 50.48 ± 1.10 µg/mL against AChE and BChE, respectively. The compounds exhibited potential inhibition of α-amylase and α-glucosidase, especially the phenolic compounds (**1**–**5**). Myricetin-3-O-rhamnoside had the highest α-amylase inhibition with an IC_50_ value of 65.17 ± 0.43 µg/mL compared to acarbose with an IC_50_ value of 32.25 ± 0.36 µg/mL. Two compounds, 3,3′-di-O-methyl ellagic acid (IC_50_ = 74.18 ± 0.29 µg/mL) and myricetin-3-O-rhamnoside (IC_50_ = 69.02 ± 0.65 µg/mL), were more active than the standard acarbose (IC_50_ = 87.70 ± 0.68 µg/mL) in the α-glucosidase assay. For α-glucosidase and α-amylase, the molecular docking results for **1–11** reveal that these compounds may fit well into the binding sites of the target enzymes, establishing stable complexes with negative binding energies in the range of −4.03 to −10.20 kcalmol^−1^. Though not all the compounds showed binding affinities with cholinesterases, some had negative binding energies, indicating that the inhibition was thermodynamically favorable.

## 1. Introduction

Enzymes are vital in biochemical processes and play important roles in signaling pathways, metabolism, defense mechanisms, and immune responses, and many of them serve as targets for natural or synthetic drug development [1]. Enzyme activities determine the health of living organisms since overexpression or deficiencies can lead to pathologies. A common enzyme-related neurological illness, Alzheimer’s disease (AD), is characterized by a progressive and irreversible loss of memory, emotional dysfunction, diminished cognitive function, impairment, dementia, and eventually patient death [2,3]. The estimated number of patients suffering from dementia is expected to increase from 57.4 million cases globally in 2019 to 152.8 million cases by 2050 [4]. Alzheimer’s disease (AD) is a fatal dementia that, according to the cholinergic hypothesis, results from low levels of neurotransmitters caused by cholinesterase, which hydrolyze acetylcholine, making cholinesterase inhibitors suitable therapeutics [5,6]. Because of this, the treatment of AD involves the use of acetylcholinesterase (AChE) and butyrylcholinesterase (BuChE) inhibitors, such as galantamine, donepezil, and rivastigmine [7]. The inhibition of these two cholinesterase enzyme types is a necessary part of this treatment [7,8]. Cholinesterase inhibitors are being developed as a remedy against AD, and more interest is turned towards natural sources that produce diverse alkaloids, phenolic compounds, coumarins, and terpenoids with great anticholinesterase potential against both acetylcholinesterase (AChE) and butyrylcholinesterase (BChE) and are therefore potential AD drug candidates [9,10,11,12].

Diabetes is a fast-growing and deadly metabolic disorder of glucose metabolism that results from chronic hyperglycemia and high blood sugar levels. An estimated 529 million diabetic individuals were predicted in 2021, possibly expected to rise to 1.31 billion diabetic individuals by 2050 worldwide, which may eventually result in high mortalities and morbidities, driven mostly by type 2 diabetes, and demographic shifts [13]. Hyperglycemia refers to high blood glucose levels caused by metabolic disturbances affecting the pancreatic β-cells, involving type 1 diabetes mellitus (insufficient insulin synthesis by the pancreas) and type 2 diabetes mellitus (ineffective action of insulin) [14]. The inhibition of glucose digestive enzymes (α-amylase and α-glucosidase) significantly reduces blood sugar levels and serves as an important strategy in the management of type 2 diabetes [15]. Conventional drugs such as acarbose and miglitol are used in regulating blood glucose levels within safe limits and delaying uptake of post-meal glucose in the blood. Natural sources provide many effective oral α-amylase and α-glucosidase inhibitors and antidiabetic therapies and compounds, including phenolics, terpenoids, and alkaloids, which have few side effects and are cheap starting materials for antidiabetic drug development [16,17]. Considering the disturbing situations and burden that are caused by AD and diabetes for patients and the difficulty of treatment, in addition to the high cost and side effects of available drugs, medicinal plants are being searched for potential new therapies. Recently, interest in natural cholinesterase, α-glucosidase, and α-amylase inhibitors is greatly increasing because the clinically available drugs are tagged with moderate to low effectiveness and a high cost and may display undesirable side effects, such as gastrointestinal complications and cardiovascular diseases and toxicities caused by chronic administration [18,19,20]. Computational and in silico approaches are relevant strategies in verifying enzyme inhibitory activities and are usually employed in combination with in vivo and in vitro studies as an integral part of the drug discovery and development process to expedite it and reduce its steps and expenses [16,21,22].

*Terminalia macroptera* is widely used in Africa to treat a variety of illnesses: its bark is used to treat malaria, diarrhea, and dysentery [23]. A decoction of the leaves is used to treat hepatitis, ringworm, skin diseases, gastritis, colic and hypertension, fever, leprosy, tuberculosis, and diabetes [24,25,26]. Previous scientific studies on the roots, leaves, and bark of *Terminalia macroptera* have reported pharmacological properties such as antibacterial, antifungal, antiplasmodic, antitrypanosomal, leishmanicidal, antiviral, anti-helicobacter pylori, anti-inflammatory, antipyretic, analgesic, hepatoprotective, antimicrobial, antimalarial, antioxidant, and antidiabetic properties [27,28,29]. Chemical studies on *Terminalia macroptera* have led to the identification of several classes of secondary metabolites, such as flavonoids, triterpenoids, ellagic tannins, and other phenolic substances [29,30].

This work focused on the evaluation of the acetylcholinesterase, butyrylcholinesterase, α-amylase, and α-glucosidase inhibitory effects of compounds isolated from *Terminalia macrocarpa* (Combretaceae). The structure–activity relationships were evaluated through molecular docking studies.

## 2. Results

### 2.1. Isolated Compounds

**Figure 1:**
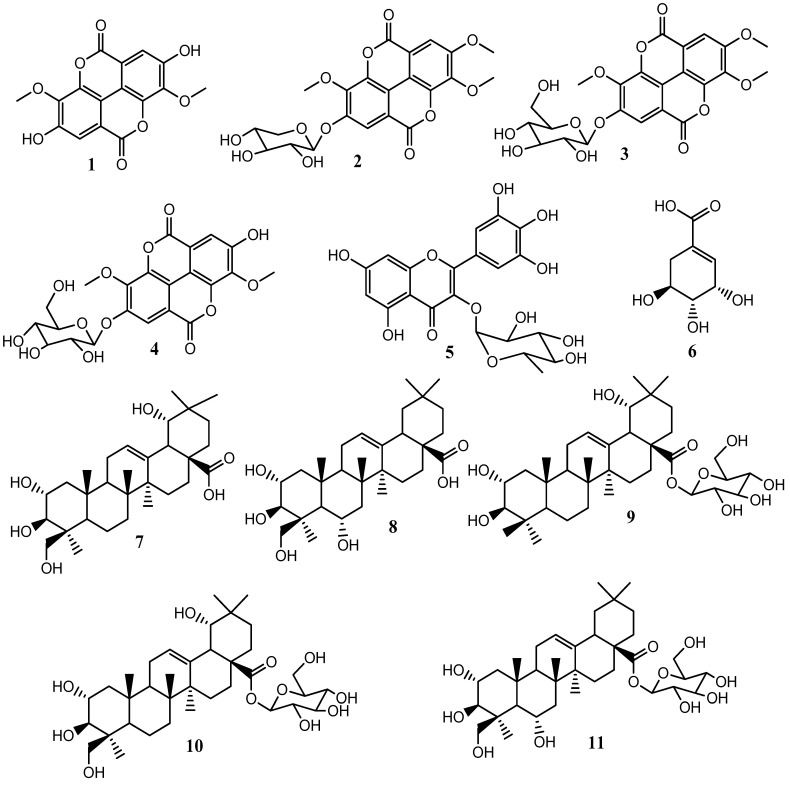
Structures of isolated compounds.

The isolated compounds were identified from their ^1^H and ^13^C NMR data and through comparison with reported spectral data as 3,3′-di-*O*-methylellagic acid (**1**), 3,3′,4′-tri-O-methylellagic acid-4-O-β-D-xylopyranoside (**2**), 3,3′,4′-tri-O-methylellagic acid-4-O-β-D-glucopyranoside (**3**), 3,3′-di-*O*-methylellagic acid-4-*O*-β-D-glucopyranoside (**4**), myricetin-3-O-rhamnoside (**5**), shikimic acid (**6**), arjungenin (**7**), terminolic acid (**8**), 24-deoxysericoside (**9**), arjunglucoside I (**10**), and chebuloside II (**11**). The structures of the isolated compounds are provided in Figure 1.

### 2.2. Anticholinesterase and Antidiabetic Effects of the Isolated Compounds

The enzyme inhibitory potential of the isolated compounds was expressed in terms of the concentration at which each compound had 50% inhibition (IC_50_), and the results are presented in Table 1. The anticholinesterase activity was evaluated up to 100 µg/mL. as concerned the α-amylase and α-glucosidase inhibitory activities, and the maximum concentration of the test compounds was 200 µg/mL. Some of the compounds showed IC_50_ values within the test concentrations, while others did not. The extract inhibited both AChE and BChE with IC_50_ values of 56.91 ± 0.47 µg/mL and 80.50 ± 1.25 µg/mL, respectively. All the derivatives of ellagic acid inhibited AChE as follows: 3,3′-di-O-methyl ellagic acid (IC_50_ = 46.77 ± 0.90 µg/mL), 3,3′,4′-tri-O-methyl ellagic acid-4-O-β-D-xylopyranoside (IC_50_ = 61.25 ± 0.11 µg/mL), 3,3′,4′-tri-O-methyl ellagic acid-4-O-β-D-glucopyranoside (IC_50_ = 59.75 ± 0.33 µg/mL), 3,3′-di-O-methyl ellagic acid-4-O-β-D-glucopyranoside (IC_50_ = 63.55 ± 0.72 µg/mL). Against BChE, only 3,3′-di-O-methyl ellagic acid (IC_50_ = 50.48 ± 1.10 µg/mL), 3,3′,4′-tri-O-methyl ellagic acid-4-O-β-D-glucopyranoside (IC_50_ = 75.21 ± 0.31 µg/mL), and 3,3′-di-O-methyl ellagic acid-4-O-β-D-glucopyranoside (IC_50_ = 89.81 ± 0.43 µg/mL) showed IC_50_ values within the tested concentrations. Shikimic acid inhibited AChE and BChE with IC_50_ values of 55.25 ± 0.25 µg/mL and 58.70 ± 0.46 µg/mL, respectively. Amongst the triterpenoids, IC_50_ values for AChE inhibition were within the tested concentrations only for arjungenin (IC_50_ = 53.61 ± 0.81 µg/mL), terminolic acid (IC_50_ = 65.72 ± 0.33 µg/mL), and arjunglucoside I (IC_50_ = 98.71 ± 1.02 µg/mL). The results can be considered to range from low to moderate when compared to the standard galantamine, with IC_50_ values of 5.50 ± 0.25 µg/mL and 42.10 ± 0.15 µg/mL against AChE and BChE, respectively.

The extracts inhibited both the carbohydrate digestive enzymes α-amylase and α-glucosidase with IC_50_ values of 164.71 ± 1.12 µg/mL and 148.20 ± 1.00 µg/mL, respectively. The compounds equally inhibited the diabetic enzymes, especially the phenolic compounds, that is, compounds **1**–**5**. For the phenolic compounds, the IC_50_ values for the α-amylase inhibitions varied from 65.17 ± 0.43 µg/mL for myricetin-3-O-rhamnoside, the most active, to 110.13 ± 0.80 µg/mL for 3,3′,4′-tri-O-methyl ellagic acid-4-O-β-D-glucopyranoside, the least active. A similar trend was observed for the α-glucosidase inhibitory effect, with the IC_50_ values ranging from 69.02 ± 0.65 µg/mL for myricetin-3-O-rhamnoside, the most active, to 137.51 ± 0.81 µg/mL for 3,3′,4′-tri-O-methyl ellagic acid-4-O-β-D-glucopyranoside, the least active. For the triterpenoids, arjungenin and terminolic acid had IC_50_ values of 186.32 ± 0.21 µg/mL and 145.91 ± 0.56 µg/mL against α-amylase, respectively. The compounds arjungenin and terminolic acid equally had IC_50_ values of 192.17 ± 0.78 µg/mL and 155.84 ± 0.31 µg/mL, respectively, against α-glucosidase. Two compounds, 3,3′-di-O-methyl ellagic acid (IC_50_ = 74.18 ± 0.29 µg/mL) and myricetin-3-O-rhamnoside (IC_50_ = 69.02 ± 0.65 µg/mL), were more active than the standard acarbose (IC_50_ = 87.70 ± 0.68 µg/mL) in the α-glucosidase assay.

### 2.3. Molecular Docking Results

The experimental anticholinesterase and antidiabetic assays of the compounds isolated from Terminalia macroptera (**1–11**) are displayed in Table 1. Compounds **1–11** belong to different families of natural compounds. From the measured and IC_50_ values, it appears that the inhibition of the isolated compounds may strongly depend on the family of compounds and the structural features of these compounds (Table 1 and Figure 1). In an attempt to explain the observed inhibitions of the isolated compounds, molecular docking was performed to determine the binding modes between each compound on one side and the active residues of α-glucosidase, α-amylase, acetylcholinesterase, and butyrylcholinesterase. Table 2 and Table 3 summarize the free binding energies, the number of hydrogen bonds, and the number of interactions in the complexes formed between the isolated compounds (**1–11**) and the active residues of the enzymes α-glucosidase, α-amylase, acetylcholinesterase, and butyrylcholinesterase.

## 3. Discussion

*T. macroptera* is an important medicinal plant in West and Central Africa whose rich chemical content has been revealed in this study, as many diversified compounds have been isolated and characterized. These compounds are phenolic compounds and pentacyclic triterpenoids. Amongst the phenolic compounds, ellagic acid derivatives (compounds **1–4**) were predominant, while the pentacyclic triterpenoids (compounds **7–11**) were all of the oleanane type. The results are comparable to previous chemical research on *T. macroptera* which described the presence of flavonoids, triterpenoids, ellagitannins, and other phenolic compounds [30,31,32,33]. Furthermore, significant derivatives of ellagic acid, together with triterpenes and saponins, are described in *T. macroptera*. This supports previous findings of some of these phyto-constituents being identified in the same plant collected from Chad [34].

The compounds showed low to moderate inhibitory effects of both cholinesterase (AChE and BChE) enzymes. Though the extract was active, some pure compounds were more active than the extract in all the assays, suggesting the absence of synergistic action amongst the compounds. However, it was observed that the derivatives of ellagic acid were generally more potent against cholinesterases when compared to the other compounds. This is possibly because the neuroprotective benefits of ellagic acid against various neurodegenerative processes have previously been described, possibly due to its mitochondrial protective properties and antioxidant and iron-chelating effects [35,36]. In a previous study, the intracerebroventricular administration of streptozotocin (STZ-ICV) in an induction model of Alzheimer’s disease was used to demonstrate that ellagic acid administration reversed a decline in cognitive functions in rat models [37]. It was observed that the non-glycosylated derivative of ellagic acid, 3,3′-di-O-methyl ellagic acid, was the most active against the cholinesterases compared to the glycosylated derivatives 3,3′,4′-tri-O-methyl ellagic acid-4-O-β-D-xylopyranoside, 3,3′,4′-tri-O-methyl ellagic acid-4-O-β-D-glucopyranoside, and 3,3′-di-O-methyl ellagic acid-4-O-β-D-glucopyranoside. This observation corroborates with the findings reported elsewhere, in which a non-glycosylated derivative of ellagic acid was more active than glycosylated ones [38]. Therefore, the presence of a sugar moiety could impede cholinesterase inhibitory effects. Shikimic acid (6) also inhibited both cholinesterases in an appreciable manner, and this may have been due to its small molecular size since it has been suggested that small molecules are more potent in terms of cholinesterase inhibition than larger ones [5]. A shikimic acid derivative with multiple sugar moieties demonstrated weak effects against the acetylcholine esterase (AChE) and butrylcholine esterase (BChE) enzymes [39]. The triterpenoids had low levels of AChE and BChE inhibition, with arjungenin, terminolic acid, and arjunglucoside I more potent than 24-deoxysericoside and chebuloside II. This suggests that the presence of a sugar moiety may reduce anticholinesterase activity. Natural triterpenoids of this class are known to possess anticholinesterase activity and are preferential to synthetic drugs [12]. Decreases in the neurotransmitter levels in the cholinergic synapses within certain regions of the brain are caused by acetylcholine and butyrylcholine hydrolysis, caused by AChE and BChE; therefore, their inhibition by these compounds is a suitable therapy for Alzheimer’s disease [40].

The phenolic compounds all exhibited inhibitory effects against the carbohydrate digestive enzymes α-amylase and α-glucosidase. α-amylase breaks down α-linked polysaccharides into smaller oligosaccharides, while α-glucosidases are membrane-bound enzymes in the small intestine that hydrolyze carbohydrate species into absorbable monosaccharides, such as glucose [41]. α-amylase and α-glucosidase inhibitors can help manage hyperglycemia as a promising approach to the management of type 2 diabetes by delaying the breakdown of carbohydrates, which lowers postprandial plasma glucose levels [42,43,44]. The ellagic acid derivatives 3,3′-di-O-methyl ellagic acid, 3,3′,4′-tri-O-methyl ellagic acid-4-O-β-D-xylopyranoside, 3,3′,4′-tri-O-methyl ellagic acid-4-O-β-D-glucopyranoside, and 3,3′-di-O-methyl ellagic acid-4-O-β-D-glucopyranoside, as well as the flavonoid myricetin-3-O-rhamnoside, exhibited moderate to good antidiabetic activity compared to the standard acarbose. The ellagic acid derivatives could be potent antidiabetic molecules, acting via various mechanisms. Some investigations report ellagic acid as a potent antidiabetic compound acting through different mechanisms, such as boasting insulin secretion and reception, the regulation of glucose transporter 4, and the mediation of several in vivo antioxidant parameters [45,46,47]. Ellagitannins and ellagic acid derivatives as well as extracts that are rich in ellagic acid have been shown to inhibit hyperglycemia and therefore could be used as anti-diabetic nutritional supplements [48]. The good antidiabetic effect of myricetin-3-O-rhamnoside is not surprising since myricetin has been shown to have a hypoglycemic effect in STZ-induced diabetic models and also as an inhibitor of the carbohydrate digestive enzymes α-glucosidase and α-amylase [49]. Two flavonoid glycosides, myricetin-3-O-rhamnoside and quercetin-3-O-rhamnoside, have displayed the capability to inhibit carbohydrate digestive enzymes and act synergistically with α-glucosidase inhibition [50]. The triterpenoids arjungenin (7) and terminolic acid (8) exhibited moderate α-amylase and α-glucosidase inhibition. The antidiabetic potential of arjungenin is corroborated by reports from previous studies. The bark extract of *T. arjuna*, which is rich in tannins, oligomeric proanthocyanidins, gallic and ellagic acids, triterpenoids, and their saponins, including arjungenin, arjunglycosides, and arjunic and arjunolic acids, has good antidiabetic activity and can stimulate the β-cells of the pancreatic islets, and its antidiabetic potential is attributable to its constituents [51]. Arjungenin and terminolic acid are constituents of *Terminalia arjuna* which have demonstrated antidiabetic and cardioprotective effects [52]. Various natural inhibitors of the AChE, BChE, α-glucosidase, and α-amylase enzymes are useful in managing ailments, especially diabetes and Alzheimer’s disease, and the search for more of such compounds is an important domain in medicinal chemistry [53].

**Figure 2:**
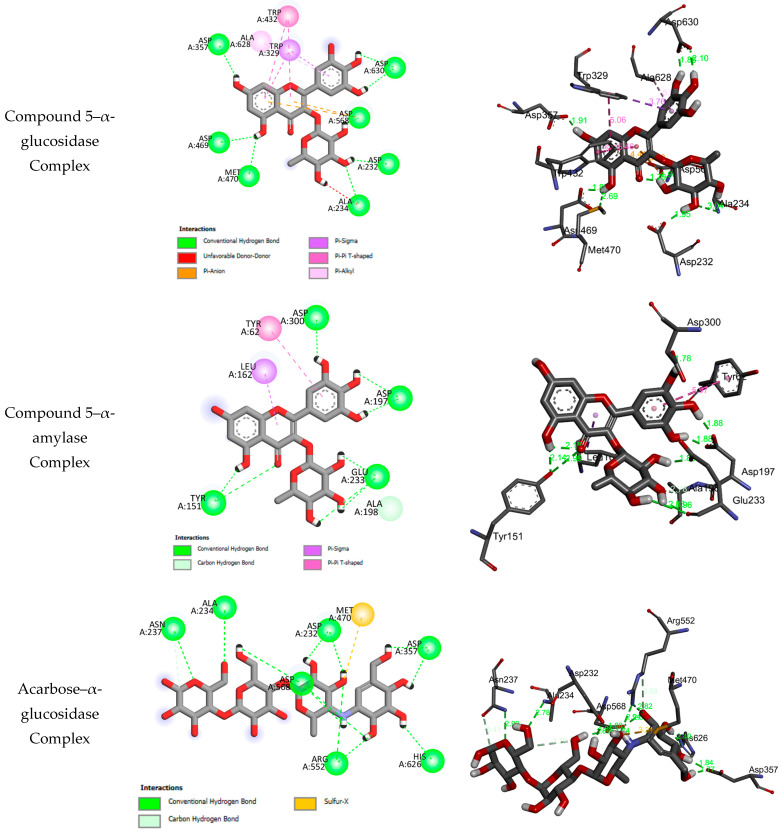

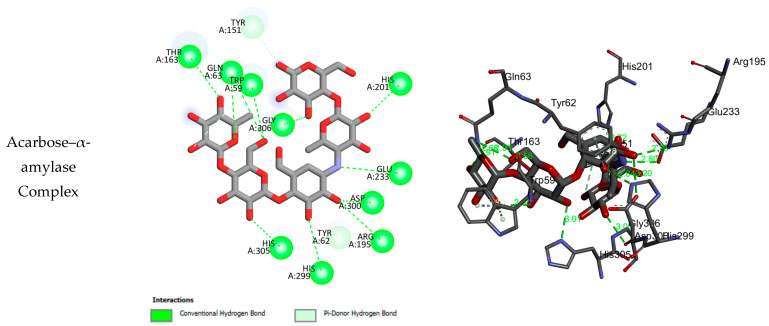
Two-dimensional and three-dimensional closest interactions of myricetin-3-O-rhamnoside (**5**) and the standard drug acarbose with the binding sites of α-glucosidase and α-amylase.

For the antidiabetic test against α-glucosidase and α-amylase, the molecular docking results for **1–11** reveal that these compounds may fit well into the binding sites of the target enzymes, and they form stable complexes with negative bending energies in the range of −4.03 to −10.20 kcalmol^−1^ (Table 1). The negative binding energies may indicate that α-glucosidase and α-amylase inhibition is a spontaneous and thermodynamically favorable process (Table 2). Compounds **1–11** may be subdivided into four subclasses, as ellagic acid derivatives (**1–4**), the flavonol myricetin-3-O-rhamnoside (**5**), shikimic acid (**6**), and pentacyclic triterpenoid derivatives (**7–11**), while each class has same basic skeleton. In the ellagic acid derivative (**1–4**) and pentacyclic triterpenoid derivative (**7–11**) subclasses, the compounds differ according to the glycosylation of hydroxyl groups. The binding interactions of **1–11** with the binding sites of α-glucosidase and α-amylase are given in Figure S1. Experimentally, the antidiabetic effects against α-glucosidase and α-amylase depend on the subclass, and for each subclass, glycosylation reduces the antidiabetic activity. Experimentally, ellagic acid derivatives show more potent antidiabetic properties compared to pentacyclic triterpenoid derivatives. However, the docking results show that glycosylation leads to the formation of stable complexes and increases the number of intermolecular hydrogen bonds with the amino acids of the target enzymes. Experimentally, the flavonol myricetin-3-O-rhamnoside (**5**) was found to have the best antidiabetic activity toward α-glucosidase and α-amylase. Figure 2 displays the binding interactions of **5** with the binding sites of α-glucosidase and α-amylase.

**Figure 3:**
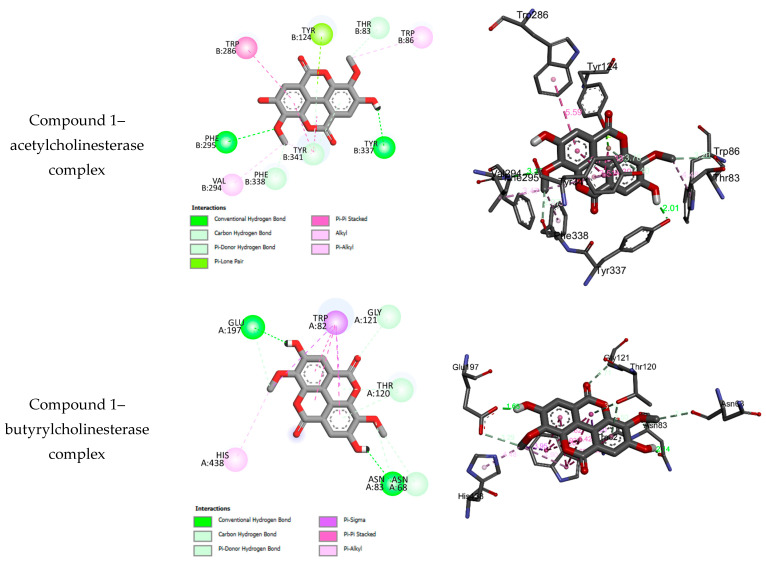

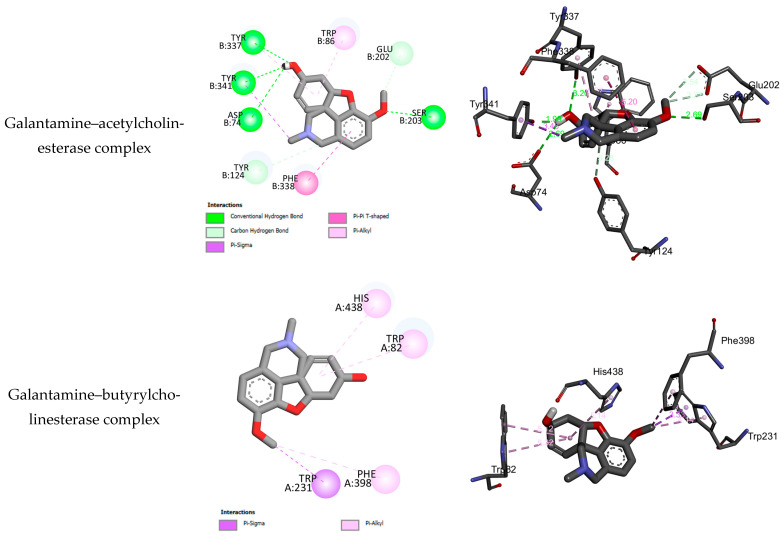
Two-dimensional and three-dimensional closest interactions of ellagic acid (**1**) and the standard drug Galantamine into the binding sites of acetylcholinesterase and butyrylcholinesterase.

For the anticholinesterase test against acetylcholinesterase and butyrylcholinesterase, the molecular docking results of **1–11** reveal that some compounds may fit well into the binding sites of the target enzymes, while some others show no binding interactions. Compounds which fit well into the binding sites of both AChE and BChE, with adequate bonding and binding energies, are suitable for the development of anticholinesterases [54]. Compounds that fit into the target enzymes form stable complexes with negative binding energies, which may indicate that the inhibition is spontaneous and thermodynamically favorable. The binding interactions of those compounds that fit into the binding sites of acetylcholinesterase and butyrylcholinesterase are given in Figure S2. Similar to the antidiabetic test, the experimental results reveal that the anticholinesterase effects against acetylcholinesterase and butyrylcholinesterase depend on the subclass of the tested compound, and for each subclass, it is suggested that glycosylation reduces the antidiabetic activity. Experimentally, ellagic acid derivatives showed stronger anticholinesterase effects compared to pentacyclic triterpenoid derivatives. However, the docking results show that glycosylation leads to the formation of stable complexes and increases the number of intermolecular hydrogen bonds with the amino acids of the anticholinesterases against acetylcholinesterase. In contrast to the antidiabetic test, the flavonol myricetin-3-O-rhamnoside (**5**) was found to be non-active. For both the acetylcholinesterase and butyrylcholinesterase targets, ellagic acid (**1**) shows the best activity. Figure 3 displays the binding interactions of compound **1** with the binding sites of acetylcholinesterase and butyrylcholinesterase.

Greater stability and binding affinity between chemical compounds and receptor proteins are typically indicated by low binding energies [55]. The prediction of ligand-binding protein interactions can be achieved using molecular docking evaluations, and this gives great insights for drug design and development. The binding affinities of isolated compounds with the amino acid residues of target proteins involved in glucose metabolism can be used to predict antidiabetic activity and help in the design of drugs to target different mechanisms of hyperglycemia [56]. In the results of molecular docking performed against the receptors of α-amylase and α-glucosidase, all the compounds interacted with the receptors to different levels, depending on the conformation of each compound inside the active site of the protein, as reflected by the docking scores, number of bonds, and binding energies. The overall results indicated the potential efficacy of the compounds in reducing blood glucose levels. For 3,3′-di-O-methyl ellagic acid (**1**) and myricetin-3-O-rhamnoside (**5**), the in silico findings did not reflect the in vitro assays. In some cases, even conventional antidiabetic drugs fail to present good binding affinities, and this may be due to the fact that they do not interact with the protein receptors efficiently in the simulations, and this could be a limitation of docking analysis [57]. Additionally, the solubility of the compounds and the experimental conditions could also create situations in which molecular docking results fail to agree with experimental results. A similar observation was made for the most active compound, 3,3′-di-O-methyl ellagic acid (**1**), in the AChE and BChE assays, which failed to exhibit the best docking scores and lowest binding energy. Ligands with good docking scores within the active sites of cholinesterase receptor proteins usually display stability and important binding affinity, capable of forming stable complexes with cholinesterases [58]. It was observed in this study that compounds **1–5** had better binding energies than the standard galantamine in the AChE assay, while compounds **1**, **2**, **3**, **4**, **5**, and **8** had lower binding energies than the reference compound galantamine in the BChE assay. No compound showed a lower binding energy than the reference compound acarbose in either the α-glucosidase or α-amylase assays. However, the binding energies of compounds **9**, **10**, and **11** were close to that of the reference molecule acarbose in the α-glucosidase assay, while the binding energies of compounds **2**, **8**, and **11** were close to that of the reference acarbose in the α-amylase assay. Since molecular docking predicts activity based on measuring the interactions of drug molecules and receptor protein enzymes and how they fit together, differences in the in vitro activities observed and the in silico results may be an indication that the molecules act through other different mechanisms.

## 4. Materials and Methods

### 4.1. Plant Material and Extraction

The *Terminalia macrocarpa* (Combretaceae) plant material was collected in Ngaoundere within the campus of the University of Ngaoundere in June 2016 and identified by Mr. Nana, a botanist at the National Herbarium of Cameroon. A voucher specimen was found in the National Herbarium of Cameroon under the reference number 47542 HNC. The wooden part of the roots of *Terminalia macrocarpa* was cut into pieces, dried, and ground into powder. The resulting powder (1.5 Kg) was extracted by maceration using a CH_2_Cl_2_–MeOH (50:50) system at room temperature and filtered after 48 h. The process was repeated three times, the filtrate was combined and evaporated under a vacuum using a rotary evaporator, and 67.5 g of the crude extract was obtained.

### 4.2. Isolation and Purification of the Compounds

Part of the extract (60 g) was used to prepare a slurry, which was fractioned on a silica gel column with a Hexane/CH_2_Cl_2_ (0–100%) system and then a CH_2_Cl_2_/MeOH (0–100%) system, in order of increasing polarity. A total of 200 fractions of equal volumes of 100 mL each were collected and then pooled into 5 major fractions (A–E) based on their TLC profiles. Fraction A was precipitated on standing and was filtered to obtain compound **1** (23.1 mg), which was soluble in pyridine. Fraction B (70 mg), obtained in the CH_2_Cl_2_/MeOH (97.5:2.5) system, was rechromatographed on a silica gel column with an isocratic CH_2_Cl_2_/MeOH (99:1) system, while collecting volumes of 100 mL each and combining them based on TLC to afford three major subfractions. Subfraction 1 was precipitated to give compound **2** (12.3 mg), and subfraction 2 gave compound **3** (9.1 mg), while subfraction 3 afforded compound **4** (19.3 mg). Compounds **2** and **3** were soluble in pyridine, while compound **4** was soluble in methanol. Fraction C (4 g), obtained in the CH_2_Cl_2_/MeOH (95:5) system, was rechromatographed on a silica gel column, and the elution was carried out using an isocratic eluent CH_2_Cl_2_/MeOH (97:3) system, and at the end of this elution, compound **10** (121 mg) and compound **11** (77 mg) were obtained as white solids which were soluble in methanol. Compounds **7** (103 mg), **8** (70 mg), and **9** (83 mg) were obtained as white solids, soluble in methanol after the purification of fraction D (6 g) using column chromatography with silica gel on the isocratic eluent system CH_2_Cl_2_/MeOH (7.5–20%) and washing the precipitates. Fraction E (1.2 g) was chromatographed twice on a silica gel column with the isocratic eluent CH_2_Cl_2_/MeOH system (60:40), followed by filtration on Sephadex LH-20 with MeOH to give compound **5** (13 mg) and compound **6** (130 mg), in the form of yellow and white powders, respectively, both soluble in methanol.

### 4.3. Anticholinesterase Activity

A standard spectrophotometric method was used to measure the anticholinesterase activity using both the butyrylcholinesterase (BChE) and acetylcholinesterase (AChE) enzymes, as described elsewhere but with slight modifications [59,60]. AChE (electric eel source) and BChE (horse serum source) were employed. The substrates used in the AChE and BChE assays were acetylthiocholine iodide and butyrylthiocholine chloride, respectively. DTNB (5,5′-Dithio-bis(2-nitrobenzoic) acid) was used for monitoring the cholinesterase activity. The reference compound used was galantamine, and the results were expressed as the 50% inhibition concentration (IC_50_) and the percentage of inhibition at the highest test concentration.

### 4.4. α-Glucosidase and α-Amylase Inhibitory Assay

The α-glucosidase inhibition was measured as described previously [61]. Briefly, 50 µL of each sample solution, glutathione, and 50 µL of α-glucosidase (fungal source from *Saccharomyces cerevisiae*) were mixed with 50 µL of PNPG (4-N-nitrophenyl-α-D-glucopyranoside) in phosphate buffer (0.01 M; pH 6.8) in a 96-well microplate and incubated at 37 °C for 15 min. Then, 50 μL of sodium carbonate solution (0.2 M) was added to quench the reaction, and the absorbances were recorded at 400 nm. The results were expressed as the 50% inhibition concentration (IC_50_) and the percentage of inhibition at the highest test concentration.

In the α-amylase inhibition assay, the starch iodine protocol was used with few changes [62,63]. A porcine pancreas source of α-amylase (50 µL), 25 µL of the samples, 6 mM NaCl, and phosphate buffer (pH 6.9) solution were mixed in a 96-well microplate and incubated at 37 °C for 10 min. Then, 50 µL of 0.05% starch solution was added to the reaction mixture and further incubated under the same conditions, after which Lugol (100 µL) and HCl (0.1 M, 25 µL) solutions were added to complete the reaction. The absorbances were then recorded at 630 nm and the results expressed as the 50% inhibition concentration (IC_50_) and the percentage of inhibition at the highest test concentration. The reference compound used was acarbose in both assays.

### 4.5. Molecular Docking Details

The binding affinities of the isolated constituents from *Terminalia macrocarpa* to the binding sites of *α*-glucosidase, *α*-amylase, acetylcholinesterase, and butyrylcholinesterase were determined using the AutoDock4 package, as reported elsewhere [22]. The X-ray coordinates of the targeted *α*-glucosidase, *α*-amylase, acetylcholinesterase, and butyrylcholinesterase and their corresponding original docked ligands were downloaded from the RCSB data bank website with the PDB codes 3W37, 1B2Y, 4EY7, and 1P0I, respectively. The molecular geometries of the isolated constituents were minimized using Merck molecular force field 94 (MMFF94) level44, and they were saved as PDB files. The binding interactions between the docked compounds with the binding sites of the target enzymes were visualized using the Discovery Studio Client (Discovery Studio Client is a product of Accelrys Inc., San Diego, CA, USA).

### 4.6. Statistical Analysis

The experiments were repeated thrice, and the results expressed as means ± SEM (standard error of mean). Significant differences between means were analyzed using Student’s t-test, and values of *p* < 0.05 were considered significant.

### 4.7. NMR Data of the Isolated Compounds

**3,3′-di-*O*-methyl ellagic acid (1)**. White solid. **^1^H NMR** (CD_3_OD, 500 MHz): *δ*_H_ ppm 7.58 (2H, *s*, H-5, H-5′) and 4.12 (3H, *s*, 3-OCH_3_, 3′-OCH_3_). **^13^C NMR** (CD_3_OD, 125 MHz): *δ*_C_ 158.8 (2COO-), 154.9 (C-4, C-4′), 141.2 (C-3′, C-3), 140.2 (C-2′, C-2), 112.0 (C-1′, C-1), 111.6 (C-6′, C-6), 111.4 (C-5, C-5′) and 60.5 (3-OCH_3,_ 3′-OCH_3_).

**3,3′,4′-tri-O-methyl ellagic acid-4-O-β-D-xylopyranoside (2)**. White solid. **^1^H NMR** (pyridine-*d*_5_, 500 MHz): *δ*_H_ ppm 8.39 (1H, *s*, H-5′), 7.82 (1H, *s*, H-5), 5.79 (1H, *d*, *J* = 8, 7 Hz, H-1″), 4.39 (1H, *m*, Hb-5″), 4.37 (1H, *m*, H-3″), 4.31 (1H, *m*, H-2″), 4.31 (1H, *m*, H-4″), 4.27 (3H, *s*, 3′-OCH_3_), 4.13 (3H, *s*, 3-OCH_3_), 3.89 (1H, *m*, Ha-5″), 3.85 (3H, *s*, 4-OCH_3_). **^13^C NMR** (pyridine-*d*_5_, 125 MHz): *δ*_C_ 158.8 (COO-), 158.7 (COO-), 154.9 (C-4), 152.7 (C-4′), 142.8 (C-3′), 141.9 (C-3), 141.8 (C-2′), 141.7 (C-2), 114.2 (C-1′), 113.9 (C-5′), 113.3 (C-1), 113.2 (C-6), 112.2 (C-6′), 108.8 (C-5), 103.7 (C-1″) 77.9 (C-2″), 74.3 (C-3″), 70.4 (C-4″), 67.1 (C-5″), 61.6 (3′-OCH_3_), 61.3 (3-OCH_3_), 56.7 (4-OCH_3_).

**3,3′,4′-tri-O-methyl ellagic acid-4-O-β-D-glucopyranoside (3)**. White solid. **^1^H NMR** (CD_3_CD, 500 MHz): *δ*_H_ ppm 7.84 (1H, *s*, H-5′), 7.60 (1H, *s*, H-5), 5.18 (1H, *d*, *J* = 7, 2 Hz, H-1″), 3.72 (1H, *m*, H_a_-6″), 3.53 (1H, *m*, H_b_-6″), 3.45 (1H, *m*, H-2″), 3.38 (1H, *m*, H-4″), 3.37 (1H, *m*, H-3″), 3.25 (1H, *m*, H-5″), 4.12 (3H, *s*, 3′-OCH_3_), 4.06 (3H, *s*, 3-OCH_3_), 4.01 (3H, *s*, 4-OCH_3_). **^13^C NMR** (CD_3_OD, 125 MHz): *δ*_C_ 158.5 (COO-), 158.1 (COO-), 154.7 (C-4), 152.3 (C-4′), 142.2 (C-3′), 141.6 (C-3), 141.6 (C-2′), 141.3 (C-2), 114.4 (C-1′), 113.2 (C-5′), 112.9 (C-1), 112.7 (C-6), 112.5 (C-6′), 108.0 (C-5), 101.7 (C-1″), 77.7 (C-5″), 76.0 (C-3″), 73.1 (C-2″), 69.9 (C-4″), 61.8 (C-6″), 61.0 (3′-OCH_3_), 61.3 (3-OCH_3_), 57.2 (4-OCH_3_).

**3,3′-di-*O*-methyl ellagic acid-4-*O*-β-D-glucopyranoside (4)**. White solid. **^1^H NMR** (CD_3_OD, 500 MHz): *δ*_H_ ppm 7.38 (1H, *s*, H-5′), 7.08 (1H, *s*, H-5′), 5.96 (1H, *d*, *J* = 8, 7 Hz, H-1″), 3.64 (3H, *s*, 3′-OCH_3_), 3.61 (3H, *s*, 3-OCH_3_), 3.26 (1H, *m*, H_a_-6″), 3.06 (1H, *m*, H_b_-6″), 2.98 (1H, *m*, H-2″), 2.97 (1H, *m*, H-4″), 2.92 (1H, *m*, H-3″), 2.92 (1H, *m*, H-5″). **^13^C NMR** (D_3_OD, 125 MHz): *δ*_C_ 158.4 (COO-), 158.3 (COO-), 152.9 (C-4), 151.7 (C-4′), 141.8 (C-3′), 141.6 (C-3), 140.9 (C-2′), 140.2 (C-2), 114.1 (C-1′), 113.2 (C-6), 112.8 (C-1), 112.2 (C-6′), 111.8 (C-5′), 111.6 (C-5), 101.4 (C-1″), 77.3 (C-2″), 76.5 (C-3″), 73.3 (C-5″), 69.5 (C-4″), 61.6 (3′-OCH_3_), 60.6 (C-6″), 61.3 (3-OCH_3_).

**Myricetin-3-O-rhamnoside (5).** Yellow solid. **^1^H NMR** (CD_3_OD, 500 MHz): *δ*_H_ ppm 6.98 (2H, *s*, H-2, H-6′), 6.39 (1H, *d*, *J* = 2.1 Hz, H-8), 6.23 (1H, *d*, *J* = 2.1 Hz, H-6), 5.34 (1H, *d*, *J* = 1.7 Hz, H-1″), 4.25 (1H, *dd*, *J* = 3.3, 1.7 Hz, H-2″), 3.82 (1H, *dd*, *J* = 9.5, 3.4 Hz, H-3″), 3.55 (1H, *dd*, *J* = 9.6, 6.2 Hz, H-5″), 3.37 (1H, t, *J* = 9.6 Hz, H-5″), 0.99 (3H, *d*, *J* = 6.2 Hz, H-6) **^13^C NMR** (CD_3_OD, 125 MHz): *δ*_C_ 179.8 (C-4), 165.9 (C-7), 163.2 (C-5), 159.4 (C-9), 158.5 (C-2), 146.8 (C-3′; C-5′), 137.8 (C-4′), 137.3 (C-3), 121.9 (C-1′), 109.6 (C-2′; C-6′), 105.8 (C-10), 103.6 (C-1″), 99.8 (C-6), 94.7 (C-8), 73.3 (C-2″), 72.1 (C-5″), 72.0 (C-4″), 71.9 (C-3″), 17.6 (C-6″).

**Shikimic acid (6).** White solid. **^1^H NMR** (CD_3_OD, 500 MHz): *δ*_H_ ppm 6.82 (1H, *dd*, H-2), 4.39 (1H, *m*, H-5), 4.01 (1H, *s*, H-4), 3.70 (1H, *dd*, H-3), 2.71(1H, *dd*, H_a_-6), 2.21 (1H, *dd*, H_b_-6), **^13^C NMR** (D_3_OD, 125 MHz): *δ*_C_ 168.5 (COOH), 137.8 (C-2), 129.5 (C-1), 71.5 (C-3), 67.4 (C-4), 66.9 (C-5), 30.3 (C-6).

**Arjungenin (7)**. White solid. **^1^H NMR** (CD_3_OD, 500 MHz): *δ*_H_ ppm 5.37 (1H, t, *J* = 3.3 Hz, H-12), 3.71 (1H, *td*, *J* = 11.3, 4.4 Hz, H-2), 3.35 (1H, *d*, *J* = 9.6 Hz, H-3), 3.36 (1H, *d*, *J* = 11.2 Hz, H-24a), 3.27 (1H, *d*, *J* = 11.7 Hz, H-24b), 3.28 (1H, *d*, *J* = 3.8 Hz, H-19), 3.07 (1H, *br*, H-18), 1.33 (1H, *m*, H-5), 1.29 (3H, *s*, H-27), 1.01 (3H, *s*, H-25), 0.94 (3H, *s*, H-29), 0.97 (3H, *s*, H-30), 0.76 (3H, *s*, H-26), 0.72 (3H, *s*, H-23), 2.5–1.0 (alicyclic protons). **^13^C NMR** (CD_3_OD, 125 MHz): *δ*_C_ 181.3 (C-28), 143.1 (C-13), 123.7 (C-12), 81.2 (C-19), 77.8 (C-3), 68.3 (C-2), 66.3 (C-23), 47.7 (C-9), 47.5 (C-5), 46.1 (C-1), 45.2 (C-17), 43.7 (C-18), 42.6 (C-4), 41.3 (C-14), 39.4 (C-8), 37.9 (C-10), 34.6 (C-20), 32.6 (C-7), 28.1 (C-21), 31.9 (C-6), 28.0 (C-15), 27.3 (C-16), 25.5 (C-30), 24.1 (C-29), 24.1 (C-27), 23.6 (C-11), 18.0 (C-22), 16.6 (C-26), 16.4 (C-25), 12.6 (C-23).

**Terminolic acid (8)**. White solid. **^1^H NMR** (CD_3_OD, 500 MHz): *δ*_H_ ppm 5.30 (lH, *t*, *J* = 3.46 Hz, H-12), 4.41 (lH, *bs*, H-6), 3.75 (lH, *m*, H-2), 3.60 (lH, *d*, *J* = 11.1 Hz, H-23), 3.46 (lH, *d*, *J* = 11.1 Hz, H-23), 3.31 (lH, *d*, *J* = 9.7 Hz, H-3), 2.89 (lH, *dd*, *J* = 13.5, 3.7 Hz, H-18), 1.40 (3H, *s*, CH_3_-25), 1.08 (3H, *s*, CH_3_-24), 1.11 (3H, *s*, CH_3_-26), 1.16 (3H, *s*, CH_3_-27), 0.93 (3H, *s*, CH_3_-29), 0.96 (3H, s, CH_3_-30), 2.5–1.0 (alicyclic protons). **^13^C NMR** (125 MHz, CD_3_OD): *δ*_C_ 180.6 (C-28), 143.2 (C-13), 122.3 (C-12), 76.8 (C-3), 68.2 (C-2), 67.1 (C-6), 64.5 (C-23), 48.1 (C-1), 48.1 (C-5), 47.6 (C-9), 46.2 (C-17), 48.8 (C-19),43.3 (C-4), 42.1 (C-14), 41.3 (C-18), 39.7 (C-7), 38.3 (C-8), 37.1 (C-l0), 33.5 (C-21), 32.4 (C-29), 32.2 (C-22), 30.2 (C-20), 27.4 (C-15), 25.1 (C-27), 23.2 (C-11), 22.7 (C-30), 22.6 (C-16), 17.6 (C-25), 17.4 (C-26), 13.8 (C-24).

**24-Deoxysericoside (9).** White powder, **^1^H NMR** (CD_3_OD, 500 MHz): *δ*_H_ ppm: 5.39 (lH, *d*, *J* = 8.2 Hz, H-l′), 5.35 (lH, *t*, *J* = 3.3 Hz, H-12), 4.44 (lH, m, H_a_-6′), 4.39 (lH, *m*, H_b_-6′), 3.64 (lH, *t*, *J* = 8.90 Hz, H-4′), 3.37 (lH, *t*, *J* = 8.85 Hz, H-3′), 3.35 (lH, *t*, *J* = 8.50 Hz, H-2′), 3.32 (lH, *m*, H-2), 3.31 (lH *m*, H-5′), 3.28 (lH, *d*, *J* = 3.29 Hz, H-19), 3.07 (lH, *bs*, H-18), 2.91 (lH, *d*, *J* = 9.46 Hz, H-3), 1.31 (3H, s, CH_3_-27), 1.03 (3H, s, CH_3_-23), 1.01 (3H, *s*, CH_3_-26), 0.96 (3H, *s*, CH_3_-29), 0.95 (3H, s, CH_3_-24), 0.82 (3H, s, CH_3_-25), 0.76 (3H, s, CH_3_-30), 2.8–1.0 (alicyclic protons). **^13^C NMR** (CD_3_OD, 500 MHz): *δ*_C_ 177.3 (C-28), 144.1 (C-13), 123.4 (C-12), 94.5 (C-l′), 83.3 (C-3), 81.0 (C-19), 77.3 (C-5′), 76.59 (C-3′), 72.5 (C-2′), 69.6 (C-4′), 68.1 (C-2), 60.9 (C-6′), 54.4 (C-5), 48.4 (C-9), 47.8 (C-l), 46.6 (C-17), 45.7 (C-18), 43.6 (C-14), 41.2 (C-8), 39.5 (C-4), 39.1 (C-l0), 34.5 (C-20), 32.4 (C-7), 31.8 (C-22), 28.1 (C-23), 28.0 (C-15), 27.8 (C-21), 27.2 (C-29), 27.0 (C-16), 23.7 (C-27), 23.5 (C-30), 23.5 (C-11), 18.3 (C-6), 16.3 (C-26), 15.9 (C-24), 15.6 (C-25).

**Arjunglucoside I (10)**. White solid. **^1^H NMR **(CD_3_OD, 500 MHz): *δ*_H_ ppm 5.41 (lH, *d*, *J* = 8.2 Hz, H-1′), 5.33 (lH, *t*, *J* = 3.31 Hz, H-12), 3.82 (lH, *dd*, *J* = 12.03, 1.4 Hz, H-6′), 3.71 (lH, *td*, *J* = 12.5, 4.2 Hz, H-2), 3.70 (lH, dd, *J* = 12.1, 4.1 Hz, H-6′), 3.39 (lH, *d*, *J* = 11.1 Hz, H-23), 3.36 (lH, *d*, *J* = 11.1 Hz, H-23), 3.41 (lH, *t*, *J* = 8.7 Hz, H-3′), 3.37 (lH, *t*, *J* = 8.4 Hz, H-5′), 3.37 (lH, *t*, *J* = 8.48 Hz, H-4′), 3.36 (lH, *d*, *J* = 1.5 Hz, H-3), 3.33 (lH, *t*, *J* = 8.8 Hz, H-2′), 3.07 (lH, *brs*, H-18), 1.32 (3H, *s*, CH_3_-27), 1.04 (3H, *s*, CH_3_-25), 0.76 (3H, *s*, CH_3_-26), 0.72 (3H, *s*, CH_3_-24), 0.93 (3H, *s*, CH_3_-29), 0.96 (3H, *s*, CH_3_-30), 2.5–1.0 (alicyclic protons). **^13^C NMR** (CD_3_OD, 500 MHz): *δ*_C_ 177.8 (C-28), 144.3 (C-13), 123.7 (C-12), 94.8 (C-1′), 81.9 (C-19), 78.7 (C-5′), 77.9 (C-3′), 78.7 (C-3), 73.5 (C-2′), 70.5 (C-4′), 69.6 (C-2), 62.3 (C-6′), 66.6 (C-23), 47.9 (C-9), 47.5 (C-1), 47.6 (C-5), 46.7 (C-17), 44.1 (C-4), 41.3 (C-14), 40.9 (C-18), 40.0 (C-8), 39.2 (C-7), 38.5 (C-l0), 31.2 (C-21), 32.0 (C-29), 29.2 (C-22), 36.0 (C-20), 29.3 (C-15), 28.7 (C-27), 25.1 (C-11), 24.8 (C-30), 26.6 (C-16), 19.7 (C-6), 17.8 (C-25), 17.1 (C-26), 13.8 (C-24).

**Chebuloside II (11).** White solid. **^1^H NMR** (CD_3_OD, 500 MHz): *δ*_H_ ppm 5.39 (lH, d, *J* = 8.1 Hz, H-1′), 5.32 (lH, *t*, *J* = 3.32 Hz, H-12), 4.38 (lH, *bs*, H-6), 4.43 (lH, *dd*, *J* = 11.67, 2.5 Hz, H-23), 3.74 (lH, *m*, H-2), 3.67 (lH, *dd*, *J* = 11.67, 4.01 Hz, H-23), 3.61 (lH, *d*, *J* = 10.6 Hz, H-23), 3.43 (lH, *m*, H-3′), 3.42 (lH, *bs*, H-23), 3.36 (lH, *m*, H-5′), 3.36 (lH, *m*, H-4′), 3.33(lH, *t*, *J* = 8.51 Hz, H-2′), 3.30 (lH, *bs*, H-3), 2.89 (lH, *dd*, *J* = 12.40, 3.77 Hz, H-18), 1.40 (3H, *s*, CH_3_-25), 1.16 (3H, *s*, CH_3_-24), 1.09 (3H, *s*, CH_3_-26), 1.08 (3H, *s*, CH_3_-27), 0.96 (3H, *s*, CH_3_-29), 0.93 (3H, *s*, CH_3_-30), 25–1.0 (alicyclic protons). **^13^C NMR** (CD_3_OD, 125 MHz): *δ*_C_ 176.6 (C-28), 142.9 (C-13), 122.4 (C-12), 94.4 (C-1′), 77.2 (C-5′), 76.9 (C-3′), 76.7 (C-3), 72.5 (C-2′), 69.7 (C-4′), 68.2 (C-2), 67.1 (C-6), 64.4 (C-6′), 61.0 (C-23), 48.7 (C-1),48.0 (C-5), 47.5 (C-9), 46.6 (C-17), 46.3 (C-19), 45.8 (C-4), 43.4 (C-14), 42.1 (C-7), 40.8 (C-18), 38.5 (C-8), 37.1 (C-l0), 33.5 (C-21), 32.0 (C-29), 31.7 (C-22), 30.0 (C-20), 27.4 (C-15), 24.9 (C-27), 23.2 (C-11), 22.6 (C-30), 22.5 (C-16), 17.6 (C-25), 17.4 (C-26), 13.8 (C-24).

## 5. Conclusions

Although AD and diabetes are managed and treated with conventional drugs, medicinal plants are gaining interest as suitable alternative therapies because of their inexpensive nature and the side effects of synthetic drugs. *Terminalia macroptera* is a flowering plant species of the Combretaceae family which is used in traditional medicine for the treatment of various diseases. In this study, eleven compounds were isolated from this plant and characterized. Amongst them, ellagic acid derivatives (compounds **1**–**4**) and oleanane-type pentacyclic triterpenoids (compounds **7**–**11**) were predominant. The compounds were evaluated for their AChE, BChE, α-amylase, and α-glucosidase inhibitory potential. The compounds showed low to moderate α-amylase inhibition, while their cholinesterase and α-glucosidase inhibition ranged from moderate to good. The results indicate that the compounds could be used to develop therapies against AD and diabetes. Structure–activity relationship analysis using molecular docking studies revealed that some compounds had good interactions with low binding energies and thus could form stable complexes, with their enzyme inhibitions being spontaneous and thermodynamically favorable.

## Figures and Tables

**Table 1:** Anticholinesterase and antidiabetic activities of isolated compounds.

	Anticholinesterase Activity		Antidiabetic Activity	
	AChE	BChE	α-Amylase	α-Glucosidase
**Compound/Standard**	IC_50_ (µg/mL)	IC_50_ (µg/mL)	IC_50_ (µg/mL)	IC_50_ (µg/mL)
**Extract**	56.91 ± 0.47	80.50 ± 1.25	164.71 ± 1.12	148.20 ± 1.00
**1**	46.77 ± 0.90	50.48 ± 1.10	67.08 ± 0.42	74.18 ± 0.29
**2**	61.25 ± 0.11	>100	102.97 ± 0.73	121.51 ± 0.65
**3**	59.75 ± 0.33	75.21 ± 0.31	110.13 ± 0.80	137.51 ± 0.81
**4**	63.55 ± 0.72	89.81 ± 0.43	91.26 ± 0.55	97.75 ± 0.49
**5**	>100	>100	65.17 ± 0.43	69.02 ± 0.65
**6**	55.25 ± 0.25	58.70 ± 0.46	>200	>200
**7**	53.61 ± 0.81	>100	186.32 ± 0.21	192.17 ± 0.78
**8**	65.72 ± 0.33	>100	145.91 ± 0.56	155.84 ± 0.31
**9**	>100	>100	>200	>200
**10**	98.71 ± 1.02	>100	>200	>200
**11**	>100	>100	>200	>200
**Galantamine**	5.50 ± 0.25	42.10 ± 0.15	NT	NT
**Acarbose**	NT	NT	32.25 ± 0.36	87.70 ± 0.68

Values represent the means ± SEM of three parallel sample measurements (*p* < 0.05). NT: not tested.

**Table 2:** Free binding energies (kcal/mol), hydrogen bonding, number of closest residues to the docked isolated compounds into the binding site of α-glucosidase and α-amylase and their corresponding IC_50_ values.

No.	BEs	HBs	Number of Closest Residues to the Docked Ligand in the Active Site	IC_50_ ± SEM
***α*-glucosidase**				
1	−5.97	2	HIS A626, PHE A601, ASP A357, TRP A329, ASP A469, TRP A432, MET A470, ARG A552, ASP A568, ASPA232, ALA A234	74.18 ± 0.29
2	−7.86	6	ASP A469, ARG A552, ASP A568, ALA A234, ILE A233, ASPA232, PHE A476, TRP A432	121.51 ± 0.65
3	−7.54	7	ASP A469, ASP A357, TRP A432, PHE A476, ASP A232, ASN A237, PHE A236, ALA A234, ASP A568, ARG A552	137.51 ± 0.81
4	−7.78	5	ASP A469, ASP A357, ASP A232, ALA A234, TRP A329, PHE A601, ALA A628, ASP A568	97.75 ± 0.49
5	−7.30	8	ASP A357, ASP A469, MET A470, ALA A234, ASP A232, ASP A568, ASP A630, TRP A329, TRP A432, ALA A628	69.02 ± 0.65
6	−4.03	4	ASP A232, LYS A506, ASN A475, SER A474	>200
7	−7.27	4	ASP A630, ASP A469, ASP A232, GLU A603	192.17 ± 0.78
8	−6.71	6	ASP A232, ASP A568, MET A470, ARG A552, ASP A630, GLU A603	155.84 ± 0.31
9	−10.16	6	ARG A552, ASP A568, ASP A357, ASP A469, TRP A432	>200
10	−9.60	5	ASP A630, GLU A603, ARG A552, ASP A568, ASP A469, ASP A357	>200
11	−10.20	6	ASP A630, ASP A469, ASP A357, ASP A568, ARG A552	>200
Acarbose	−10.56	12	ASN A237, ALA A234, ASP A232, ASP A568, MET A470, ASP A357, HIS A626, ARG A552	87.70 ± 0.68
***α*-amylase**				
1	−6.22	2	TRP A58, HIS A305, TRP A59, LEU A165, HIS A101, ASP A197	67.08 ± 0.42
2	−9.41	7	TRP A59, GLN A63, ASP A197, ARG A195, GLU A133, HIS A305	102.97 ± 0.73
3	−8.84	7	TRP A59, GLN A63, GLU A233, ASP A197, ARG A195, HIS A305	110.13 ± 0.80
4	−8.72	7	TRP A59, GLN A63, GLU A233, ASP A197, ARG A195, HIS A305	91.26 ± 0.55
5	−7.83	7	ASP A197, GLU A233, ALA A198, TYR A151, LEU A162, TYR A62, ASP A300	65.17 ± 0.43
6	−4.72	4	GLU A233, ILE A235, VAL A234	>200
7	−8.78	3	ASP A300, ASP A197	186.32 ± 0.21
8	−10.18	4	TRP A59, HIS A305, LYS A200	145.91 ± 0.56
9	−8.84	7	ASP A197, TYR A151, THR A163	>200
10	−8.95	8	ASP A197, ASP A300, TYR A151, THR A163	>200
11	−9.60	7	ASP A197, ASP A300, TYR A151, THR A163, ILE A148	>200
Acarbose	−14.46	11	THR A163, GLN A63, TRP A59, GLY A306, TYR A151, HIS A201, GLU A233, ASP A300, ARG A195, TYR A62, HIS A299, HIS A305	32.25 ± 0.36

Note: In amino acid abbreviations, letter “A” refers to “A” chain of the target protein.

**Table 3:** Free binding energies, hydrogen bonding, number of closest residues to the docked isolated compounds into the binding site of acetylcholinesterase and butyrylcholinesterase and their corresponding IC_50_ values.

No.	BEs (kcal/mol)	HBs	Number of Closest Residues to the Docked Ligand in the Active Site	IC_50_ ± SEM
**Acetylcholinesterase**				
1	−8.21	2	PHE B295, VAL B295, PHE B338, TYR B341, TYR B337, TRP B86, THR B83, TYR B124, TRP B286	46.77 ± 0.90
2	−10.08	3	HIS B447, TYR B337, PHE B338, TYR B341, TRP B286, TYR B124, THR B83, TRP B86	61.25 ± 0.11
3	−10.21	3	SER B203, TYR B124, TYR B72, TRP B286, PHE B295, TYR B341, TYR B337, PHE B338	59.75 ± 0.33
4	−9.86	3	SER B203, TYR B124, TYR B72, TRP B286, PHE B295, TYR B341, PHE B338	63.55 ± 0.72
5	−8.45	5	TRP B286, TYR B341, TYR B124, ASP B74, THR B83, ASN B87, GLY B122	>100
6	−4.13	5	TYR B341, TYR B124, PHE B295, ARG B296	55.25 ± 0.25
7	−7.10	1	TRP B286, TYR B341, ARG B296, PHE B338	53.61 ± 0.81
8	−7.56	2	SER B293, TYR B341	65.72 ± 0.33
9	−6.13	3	ARG B296, TYR B341, ASP B74, TRP B286, TRP B86, PHE B338	>100
10	−5.72	2	ARG B296, PHE B338, TRP B86, TYR B341, TRP B286, ASP B74	98.71 ± 1.02
11	−6.57	5	TRP B86, ASP B74, TYR B341, TYR B72, TRP B86	>100
Galantamine	−7.59	4	ASP B74, TYR B341, TYR B337, TRP B86, GLU B202, SER B203, PHE B338, TYR B124	5.50 ± 0.25
**Butyrylcholinesterase**				
1	−7.83	2	HIS A438, ASN A83, ASN A68, THR A120, GLY A121, TRP A82, GLU A197	50.48 ± 1.10
2	−8.10	5	GLU A197, ALA A328, PHE A329, GLY A117, VAL A288, TRP A231, LEU A286, SER A198, TRP A82	>100
3	−8.04	5	LEU A286, SER A287, PRO A285, ASP A70, THR A120, GLY A115, TYR A128, LEU A125, GLU A197, GLY A116, SER A198, TRP A82	75.21 ± 0.31
4	−8.14	6	TYR A128, GLU A197, TRP A82, SER A198, LEU A286, VAL A288, SER A287, PHE A329	89.81 ± 0.43
5	−9.66	11	SER A198, GLY A116, TYR A128, GLU A197, ASP A70, TYR A332, ALA A328, HIS A438, TRP A231, LEU A286	>100
6	−5.13	4	LEU A286, PRO A285, SER A198, GLY A117	58.70 ± 0.46
7	−5.00	3	HIS A438, TRP A82, SER A287	>100
8	−6.77	3	HIS A438, TRP A82, SER A287	>100
9	NB	NA	NA	>100
10	NB	NA	NA	>100
11	NB	NA	NA	>100
Galantamine	−6.69	0	HIS A438, TRP A82, PHE A398, TRP A231	42.10 ± 0.15

Note: In amino acid abbreviations, letters “A and B” refer to “A and B” chains of the target protein.

## Data Availability

The data supporting the reported results can be obtained from the corresponding author upon reasonable request.

## Supplementary Materials

The following supporting information can be downloaded at https://www.mdpi.com/article/10.3390/molecules29112456/s1. Figure S1: Free binding energies (kcal/mol), hydrogen bonding, number of closest residues to the docked isolated compounds for the binding sites of α-glucosidase and α-amylase. Figure S2: Free binding energies, hydrogen bonding, number of closest residues to the docked isolated compounds for the binding sites of acetylcholinesterase and butyrylcholinesterase. Figure S3: ^1^H NMR and ^13^C NMR spectra of compounds **1**–**11**.
