# Supplementary material for: In Vitro and Molecular Docking Evaluation of the Anticholinesterase and Antidiabetic Effects of Compounds from Terminalia macroptera Guill. & Perr. (Combretaceae)
Source: Molecules. 2024 May 23;29(11):2456. doi: 10.3390/molecules29112456 (PMC11174011; doi:10.3390/molecules29112456)
Supplement: Supplementary file 1 [file molecules-29-02456-s001.zip › molecules-2996967-supplementary.pdf]

In vitro and molecular docking evaluation of anticholinesterase and antidiabetic effects of compounds from *Terminalia macroptera* Guill. & Perr. (Combretaceae)

Romeo Toko Feunaing [1], Alfred Ngenge Tamfu [2,3,4,5,*], Abel Joel Yaya Gbaweng [1], Selcuk Kucukaydin [3,4], Joseph Tchamgoue [6,7], Alain Meli Lannang [2], Bruno Ndjakou Lenta [6], Simeon Fogue Kouam [6], Mehmet Emin Duru[4], El Hassane Anouar [8], Emmanuel Talla [1,2], Rodica Mihaela Dinica [5,*]

1 Department of Chemistry, Faculty of Sciences, University of Ngaoundere, P.O. Box 454, Ngaoundere, Cameroon
2 Department of Chemical Engineering, School of Chemical Engineering and Mineral Industries, University of Ngaoundere, P.O. Box 454 Ngaoundere, Cameroon.
3 Department of Medical Services and Techniques, Koycegiz Vocational School of Health Services, Mugla Sitki Kocman University, Mugla, 48800, Turkey
4 Department of Chemistry, Faculty of Science, Mugla Sitki Kocman University, 48000 Mugla, Turkey
5 Department of Chemistry, Physics and Environment, Faculty of Sciences and Environment, 'Dunarea de Jos University', Galati, 47 Domneasca Str., 800008, Galati, Romania
6 Department of Chemistry, Higher Teacher Training College, The University of Yaoundé 1, P.O. Box 47, Yaoundé, Cameroon
7 Department of Organic Chemistry, Faculty of Science, University of Yaounde 1, .O. Box 812, Yaoundé, Cameroon
8 Department of Chemistry, College of Sciences and Humanities in Al-Kharj, Prince Sattam bin Ab-dulaziz University, Al-Kharj, Saudi Arabia
* Correspondence: macntamfu@yahoo.co.uk (A.N.T.); rodica.dinica@ugal.ro (R.M.D.); Tel.: +237-675590353 (A.N.T.); +33-6130-251 (R.M.D.)

Abstract: Abstract: Alzheimer's disease (AD) and diabetes are non-communicable diseases with global impacts. Inhibitors of acetylcholinesterase (AChE) and butyrylcholinesterase (BChE) are suitable therapies for AD, while α-amylase and α-glucosidase inhibitors are employed as antidiabetic agents. Compounds were isolated from the medicinal plant *Terminalia macroptera* and evaluated for their AChE, BChE, α-amylase and α-glucosidase inhibitions. From ^{1}H and ^{13}C NMR data, the compounds were identified as 3,3′-di-O-methyl ellagic acid (1), 3,3′,4′-tri-O-methyl ellagic ac-id-4-O-β-D-xylopyranoside (2), 3,3′,4′-tri-O-methyl ellagic acid-4-O-β-D-glucopyranoside (3), 3,3′-di-O-methyl ellagic acid-4-O-β-D-glucopyranoside (4), myricetin-3-O-rhamnoside (5), shi-kimic acid (6), arjungenin (7), terminolic acid (8), 24-deoxysericoside (9), arjunglucoside I (10) and chebuloside II (11). The derivatives of ellagic acid (1-4) showed moderate to good inhibition of cholinesterases with the most potent being 3,3′-di-O-methyl ellagic acid with IC_{50} of 46.77±0.90 μg/mL and 50.48±1.10 μg/mL against AChE and BChE respectively. The compounds exhibited potential inhibition of α-amylase and α-glucosidase especially the phenolic compounds (1-5). Myricetin-3-O-rhamnoside had the highest α-amylase inhibition with an IC_{50} value of 65.17±0.43 μg/mL compared to acarbose with IC_{50} of 32.25±0.36 μg/mL. Two compounds, 3,3′-di-O-methyl ellagic acid (IC_{50} = 74.18±0.29 μg/mL) and myricetin-3-O-rhamnoside (IC_{50} = 69.02±0.65 μg/mL) were more active than the standard acarbose (IC_{50} = 87.70±0.68 μg/mL) in the α-glucosidase as-say. For α-glucosidase and α-amylase, molecular docking results of 1-11 reveal that these com-pounds may fit well into the binding sites of target enzymes, establishing stable complexes with negative binding energies in the range of -4.03 to -10.20 kcalmol^{-1}. Though not all compounds showed binding affinities with cholinesterases, some had negative binding energies indicating that the inhibition is thermodynamically favorable.

Keywords: *Terminalia macroptera*; diabetes; Alzheimer's disease; cholinesterase inhibition; α-glucosidase inhibition; α-amylase inhibition; molecular

<u>List of supplementary materials</u>

Figure S1: Figure S1 Free binding energies (kcal/mol), hydrogen bonding, number of closest residues to the docked isolated compounds into the binding site of α-glucosidase, and α-amylase.
Figure S2: Figure S2. Free binding energies, hydrogen bonding, number of closest residues to the docked isolated compounds into the binding site of acetylcholinesterase, and butyrylcholinesterase.
Figure S3: ^{1}H NMR and ^{13}C NMR spectra of compounds 1-11.

Figure S1 Free binding energies (kcal/mol), hydrogen bonding, number of closest residues to the docked isolated compounds into the binding site of α-glucosidase, and α-amylase.

No.	BEs	HBs	2D Interactions	3D Interactions
1	-5.97	2	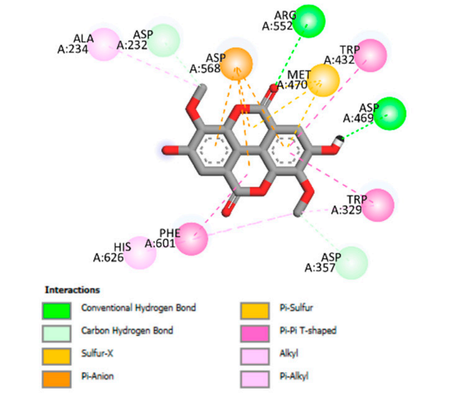	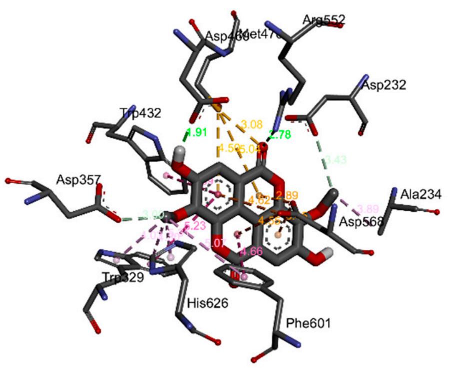
2	-7.86	6	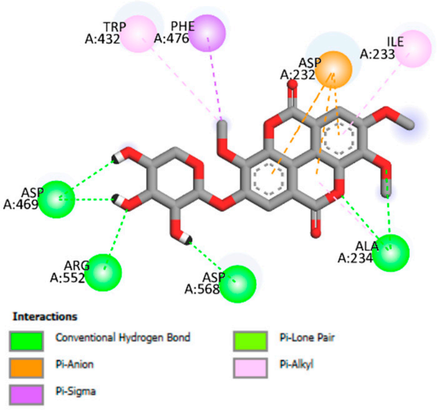	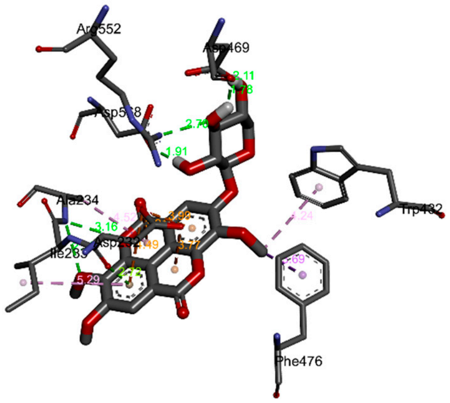

3	-7.54	7	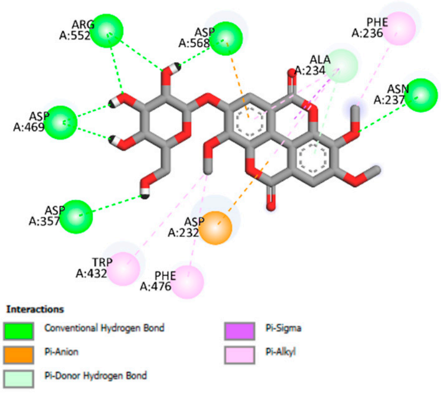	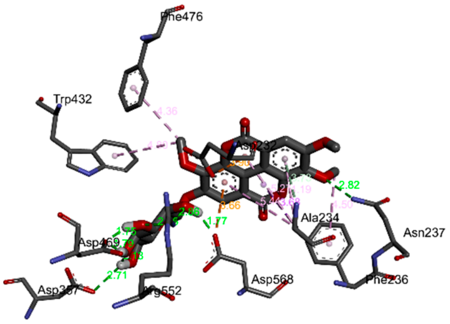
4	-7.78	5	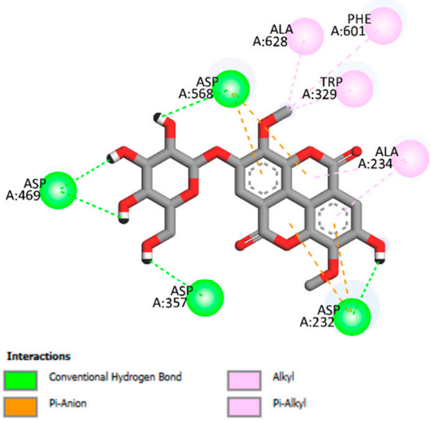	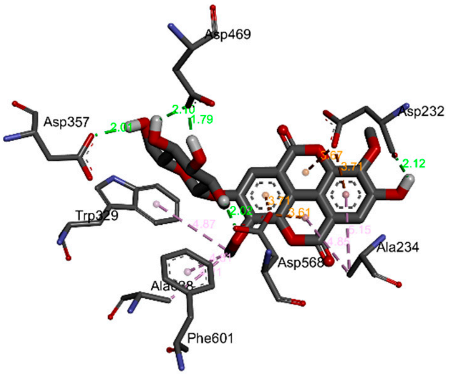
5	-7.30	8	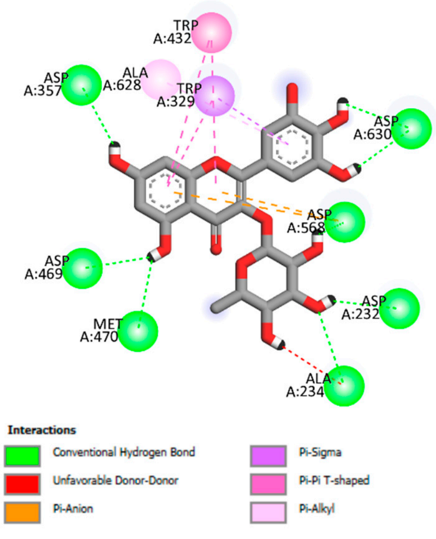	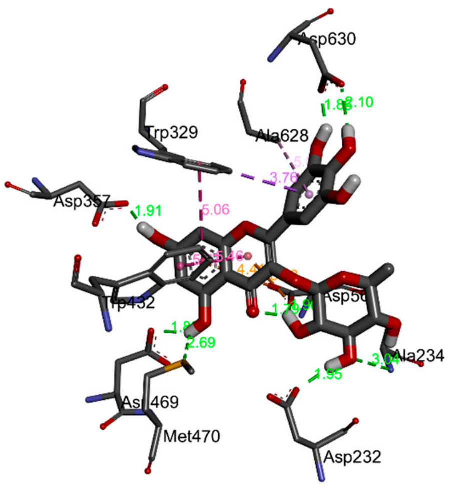

6	-4.03	4	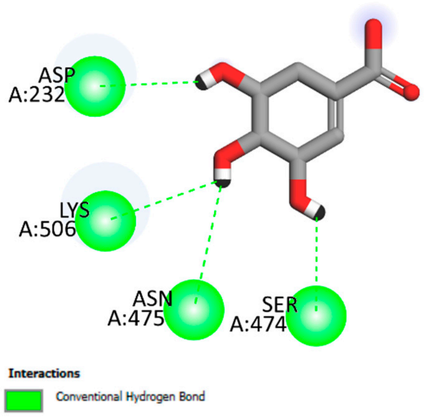	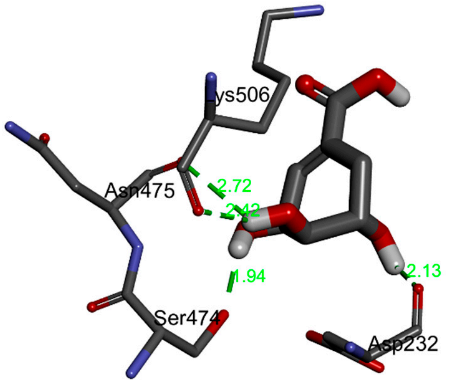
7	-7.27	4	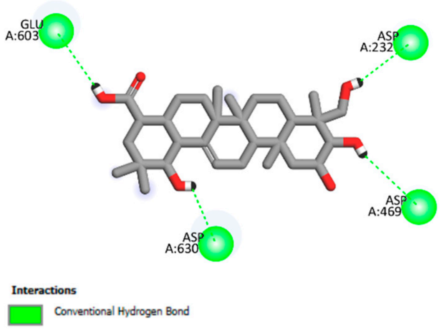	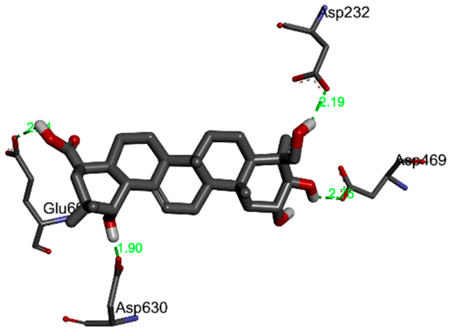
8	-6.71	6	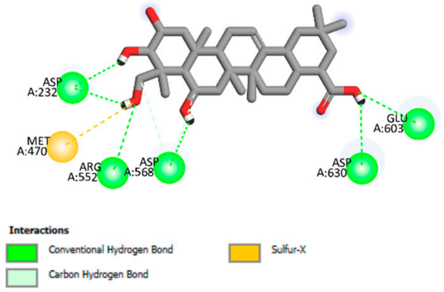	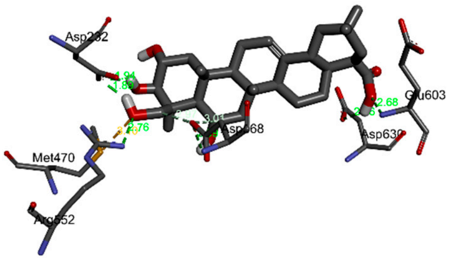
9	-10.16	6	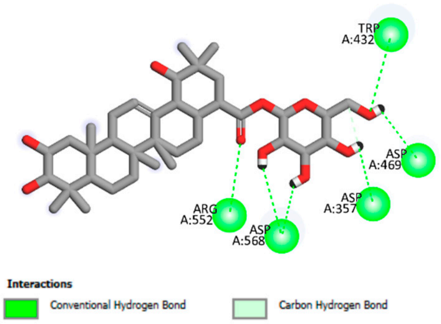	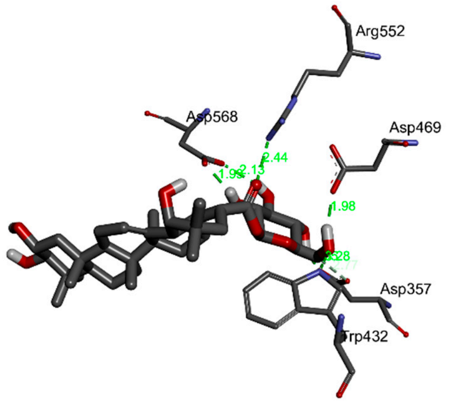

<table>
<tr><td>10</td><td>-9.60</td><td>5</td><td>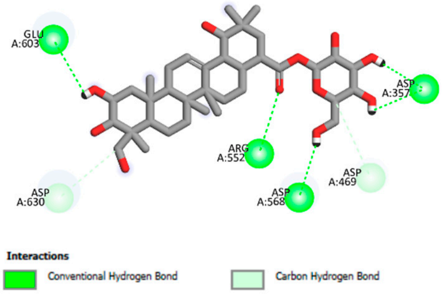
</td><td>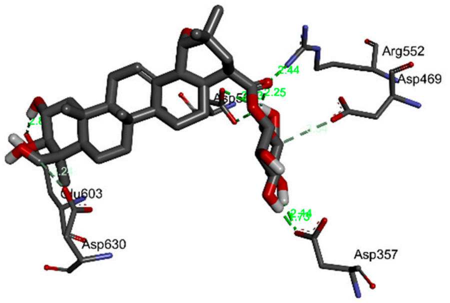
</td></tr>
<tr><td>11</td><td>-10.20</td><td>6</td><td>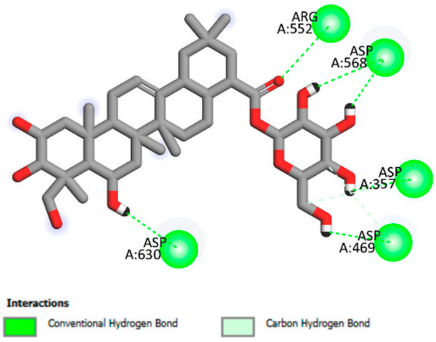
</td><td>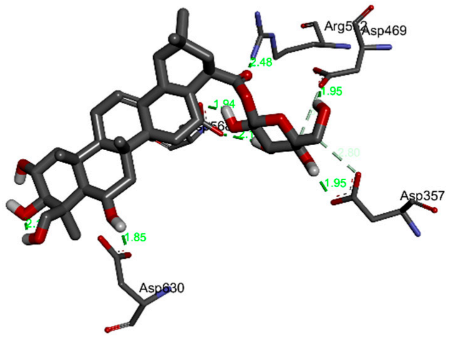
</td></tr>
<tr><td></td><td></td><td></td><td></td><td></td></tr>
<tr><td>1</td><td>-6.22</td><td>2</td><td>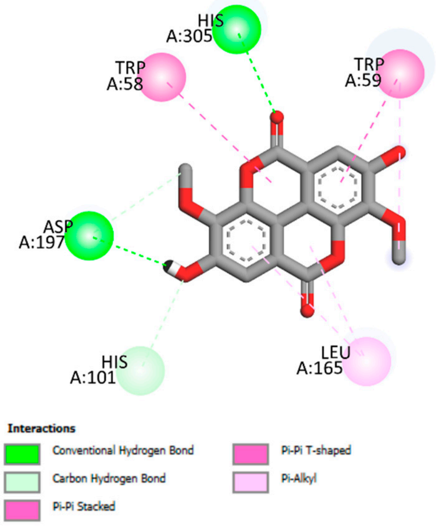
</td><td>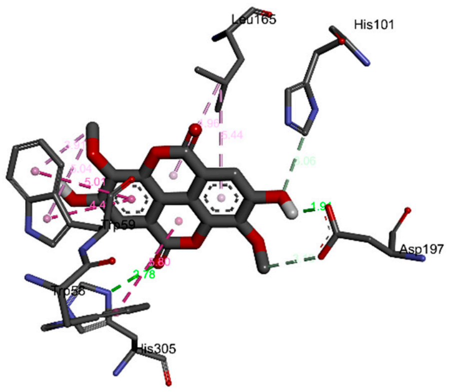
</td></tr>
<tr><td>2</td><td>-9.41</td><td>7</td><td>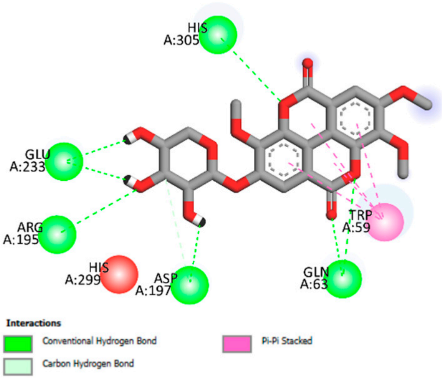
</td><td>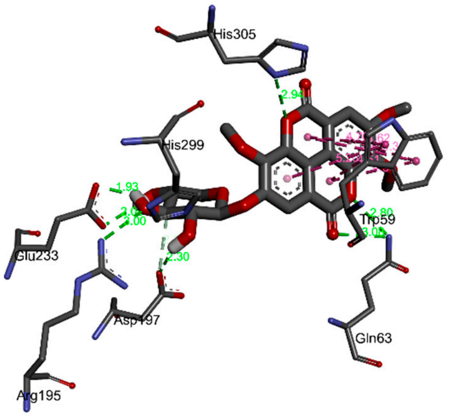
</td></tr>
</table>

3	-8.84	7	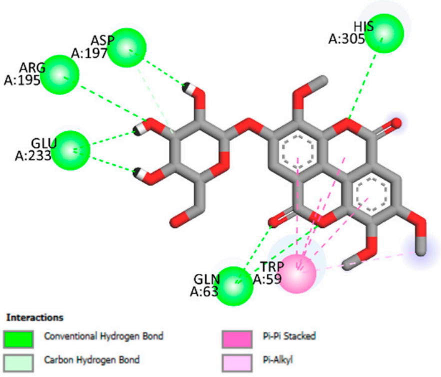	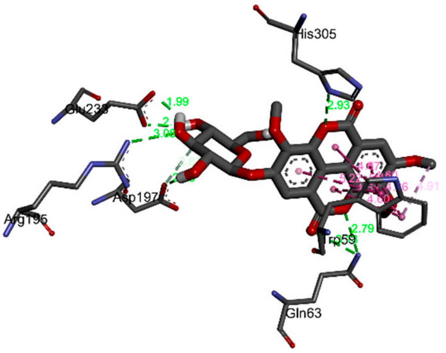
4	-8.72	7	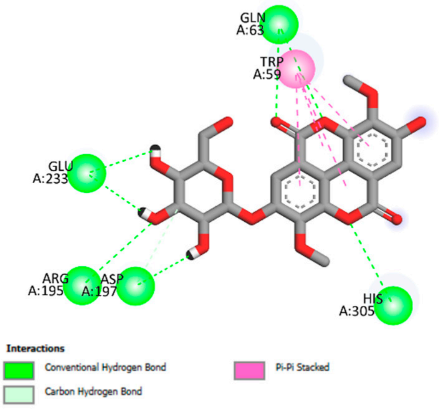	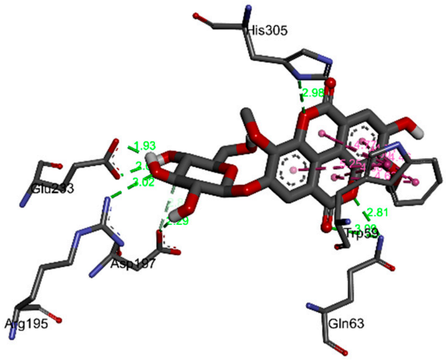
5	-7.83	7	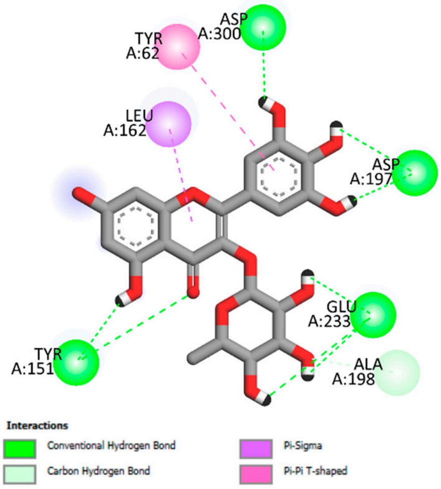	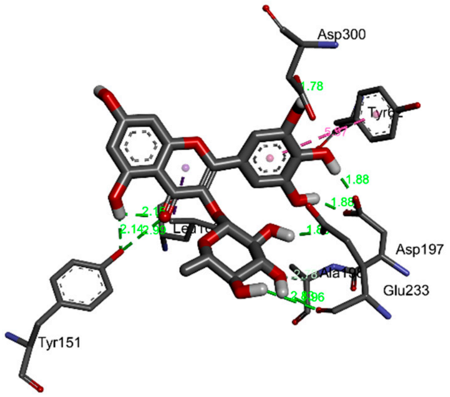

6	-4.72	4	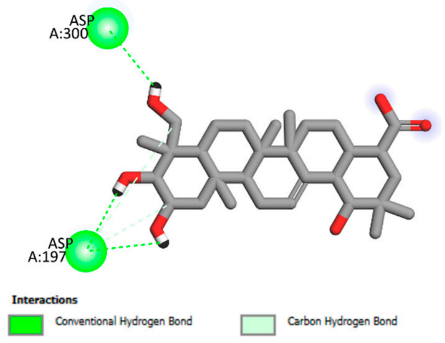	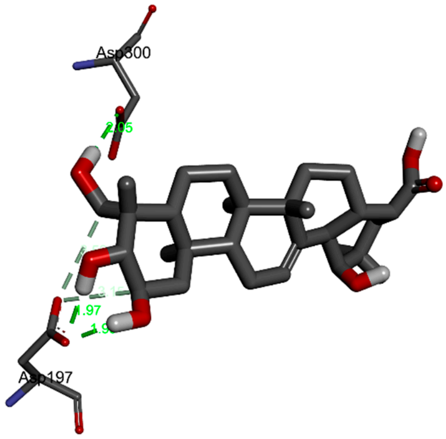
7	-8.78	3	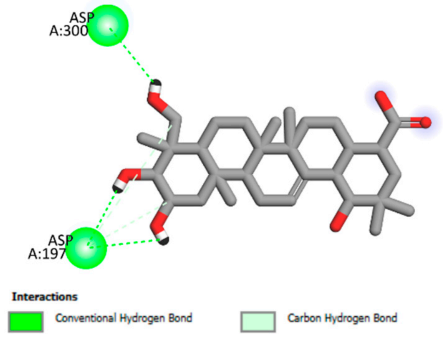	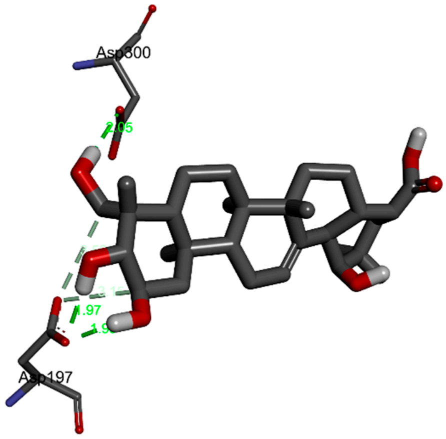
8	-10.18	4	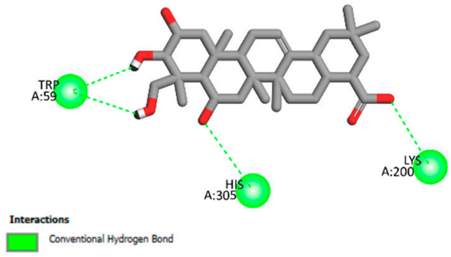	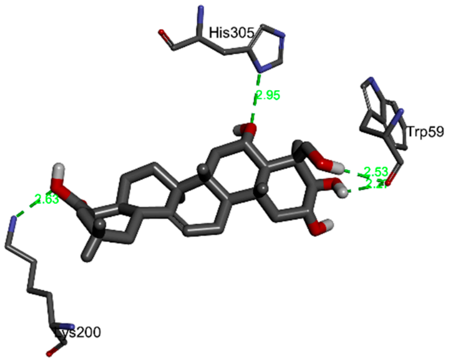
9	-8.84	7	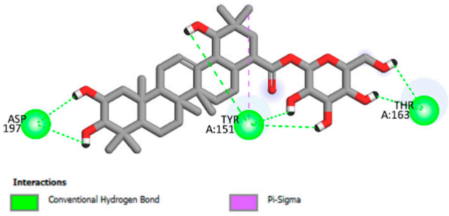	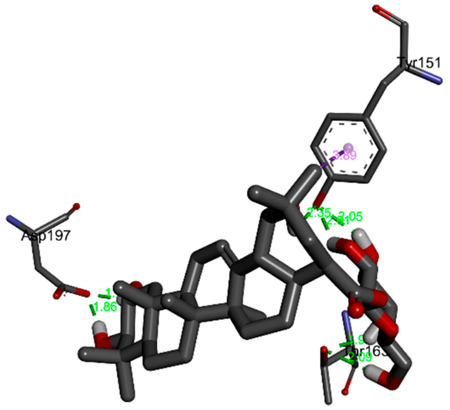

10	-8.95	8	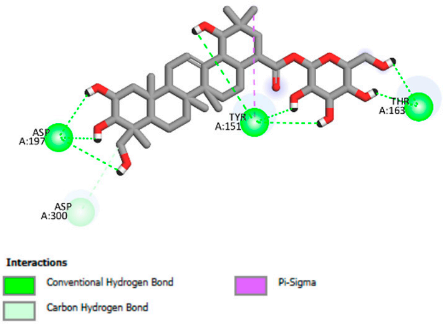	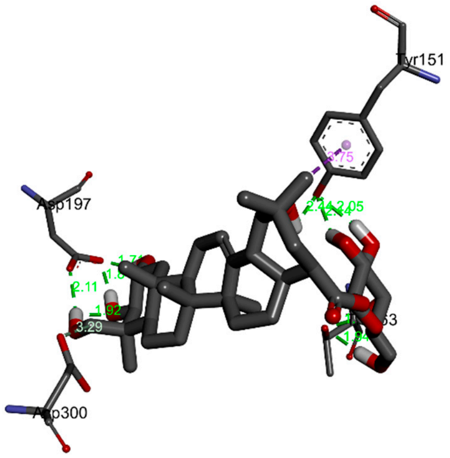
11	-9.60	7	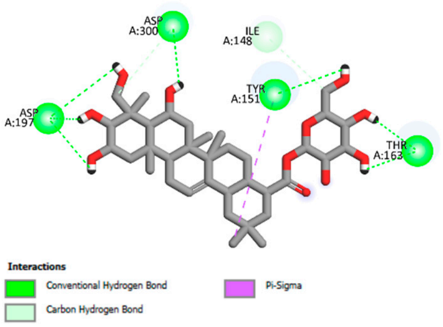	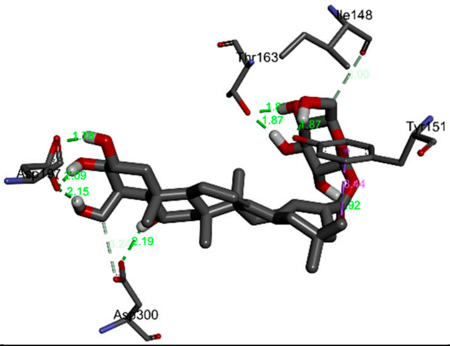

Figure S2. Free binding energies, hydrogen bonding, number of closest residues to the docked isolated compounds into the binding site of acetylcholinesterase, and butyrylcholinesterase.

No.	BEs (kcal/mol)	HBs	2D Interactions	3D Interactions
Acetylcholinesterase				
1	-8.21	2	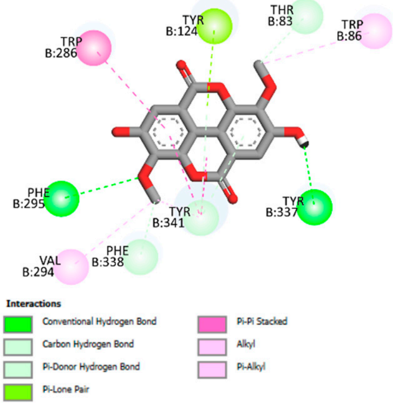	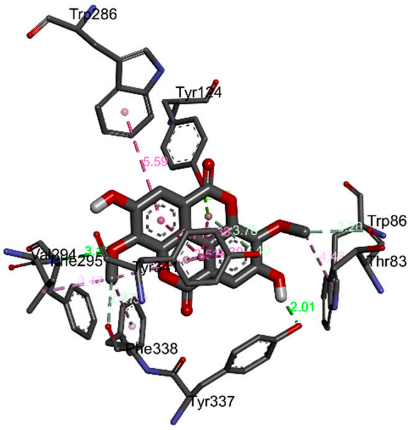

2	-10.08	3	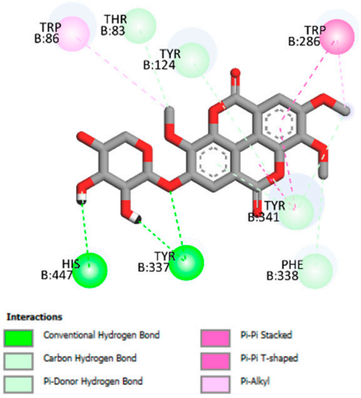	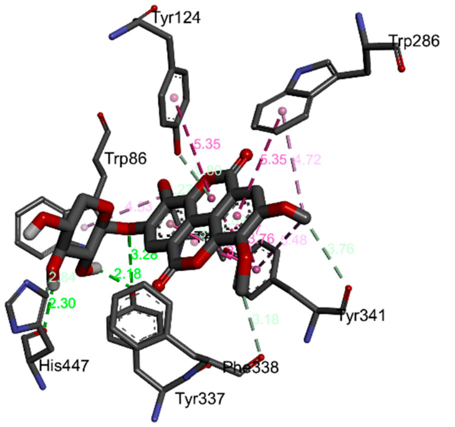
3	-10.21	3	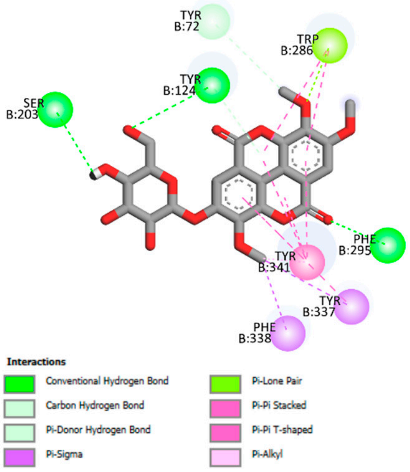	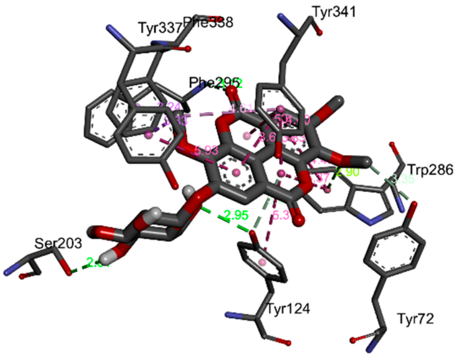
4	-9.86	3	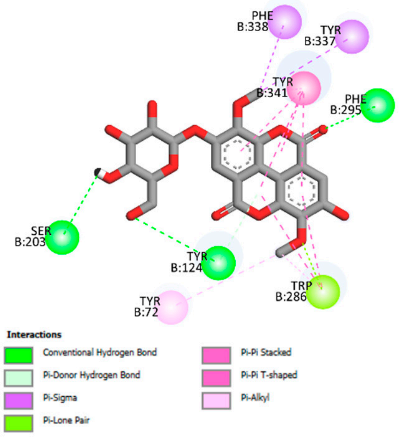	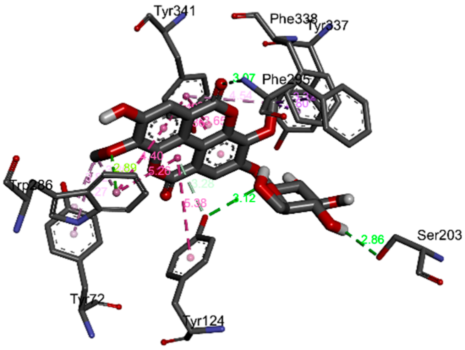

5	-8.45	5	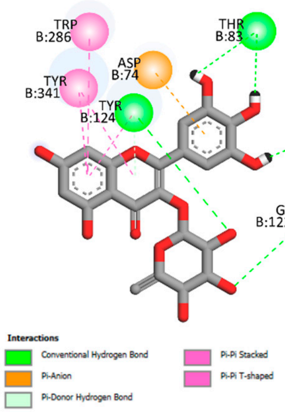	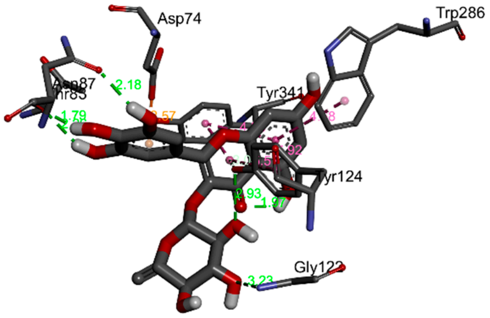
6	-4.13	5	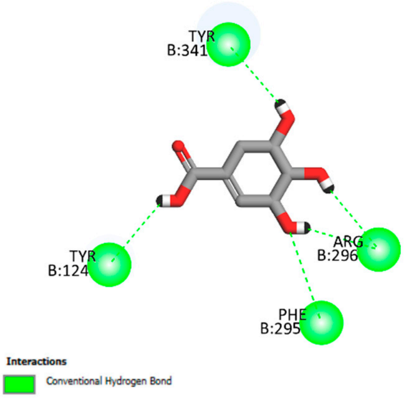	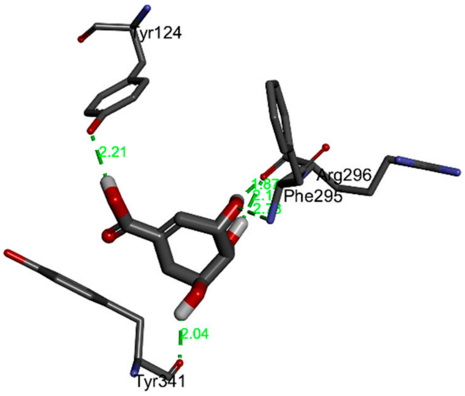
7	-7.10	1	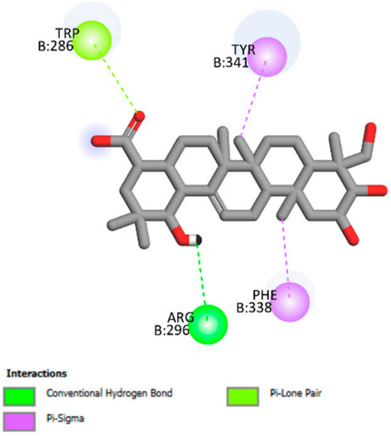	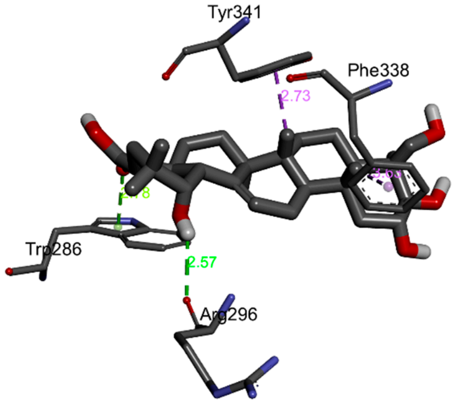

8	-7.56	2	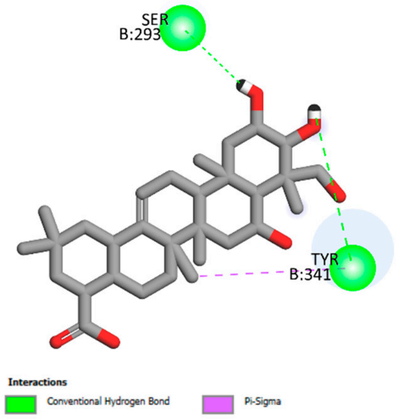	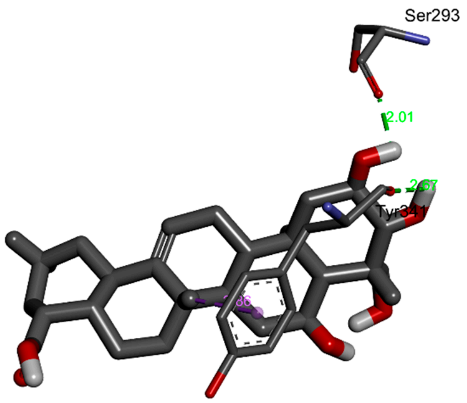
9	-6.13	3	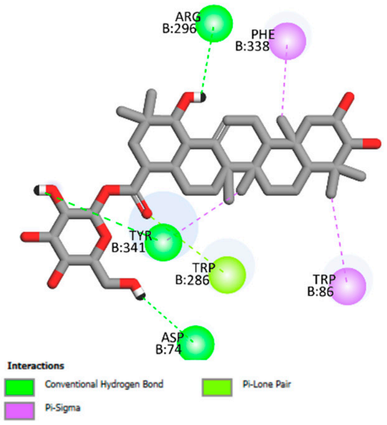	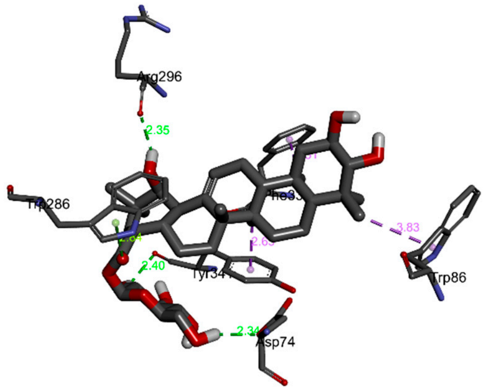
10	-5.72	2	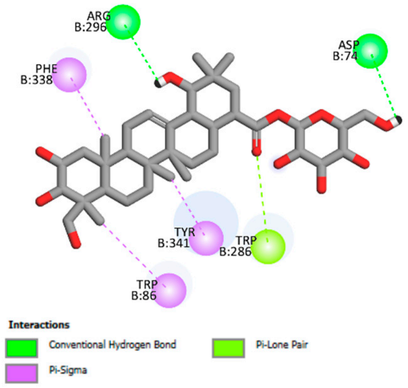	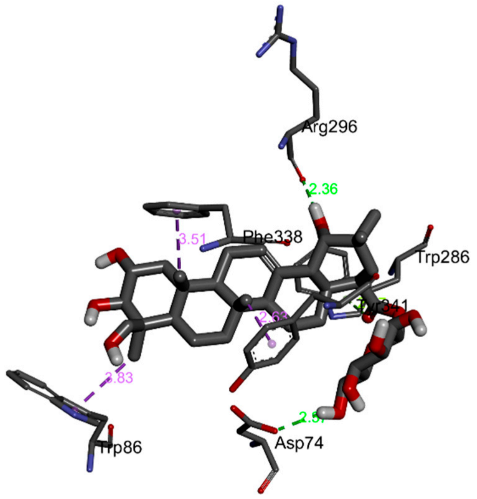

11 | -6.57 | 5

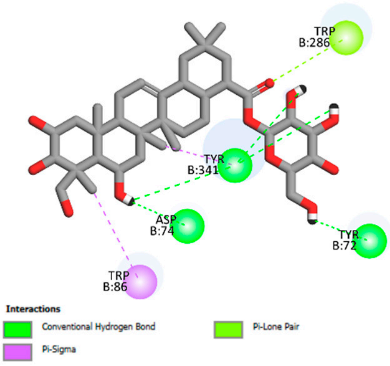

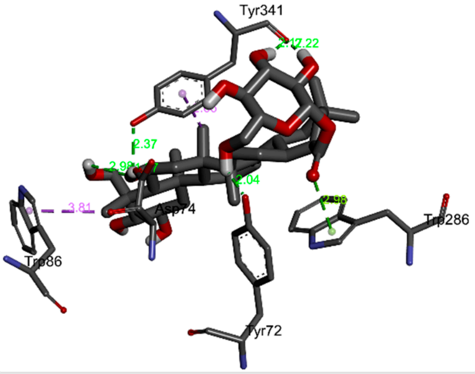

Butyrylcholinesterase

1 | -7.83 | 2

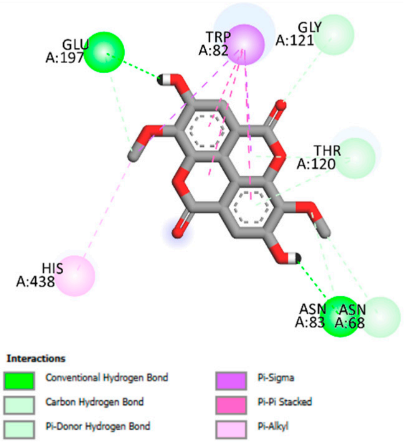

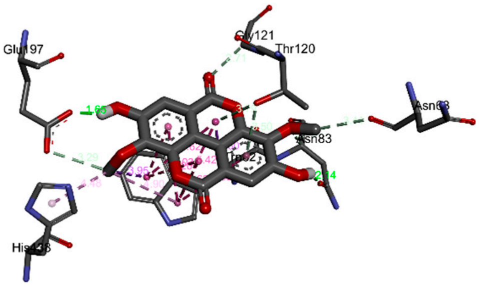

2 | -8.10 | 5

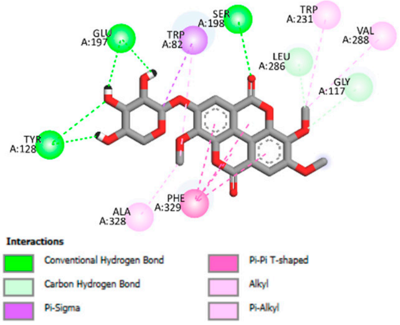

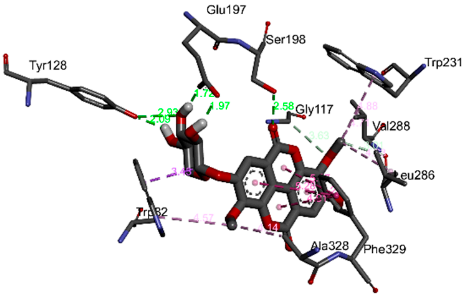

3 | -8.04 | 5

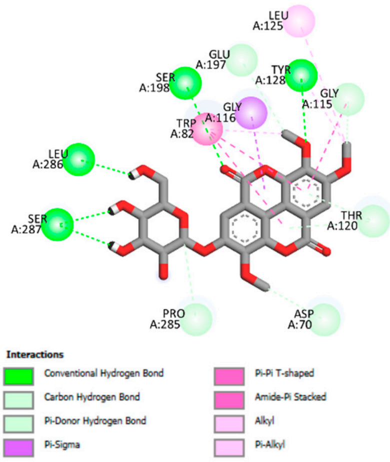

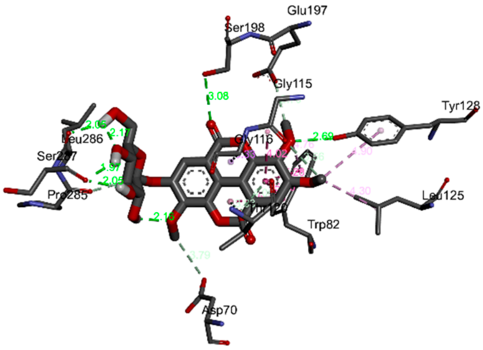

<table>
<tr><td>4</td><td>-8.14</td><td>6</td><td>
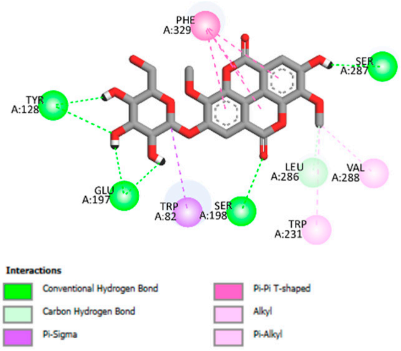

</td><td>
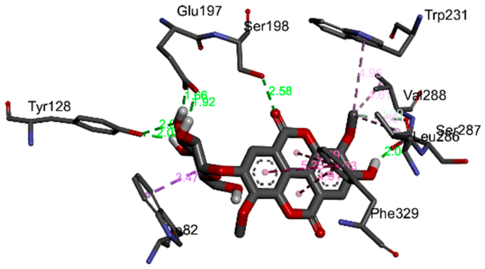

</td></tr>
<tr><td>5</td><td>-9.66</td><td>11</td><td>
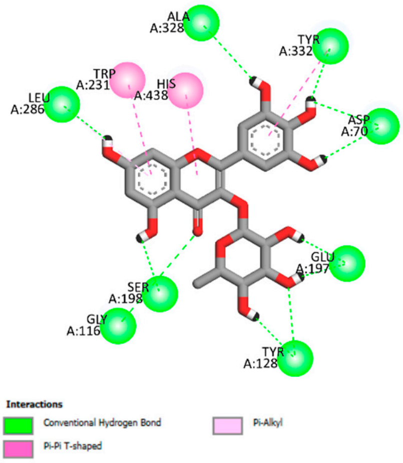

</td><td>
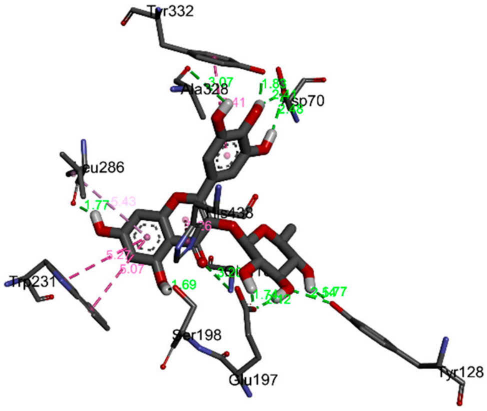

</td></tr>
<tr><td>6</td><td>-5.13</td><td>4</td><td>
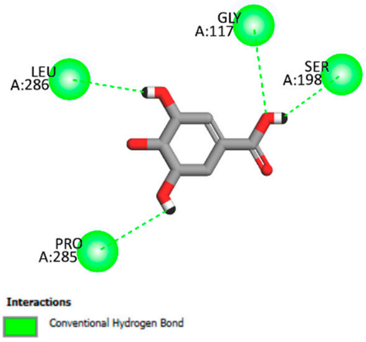

</td><td>
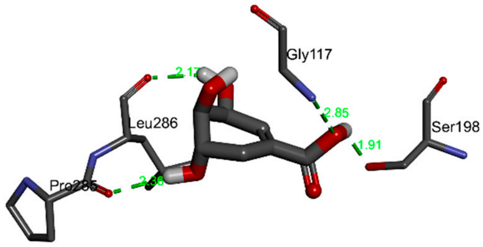

</td></tr>
<tr><td>7</td><td>-5.00</td><td>3</td><td>
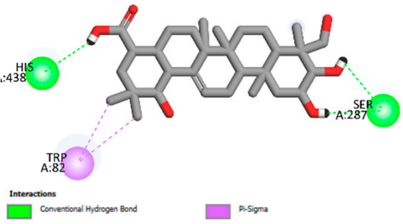

</td><td>
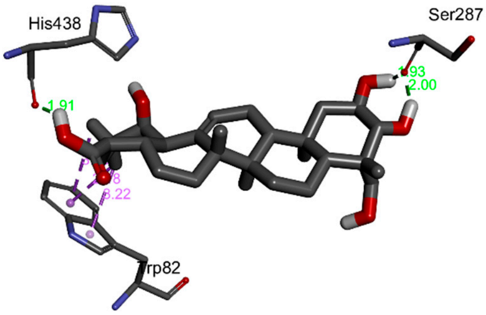

</td></tr>
</table>

8	-6.77	3	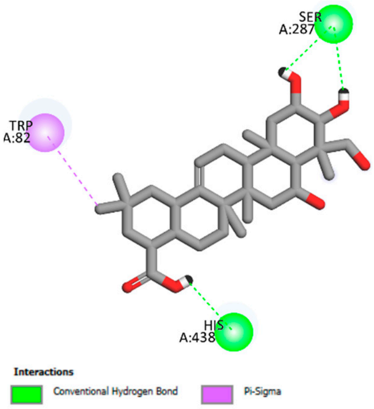	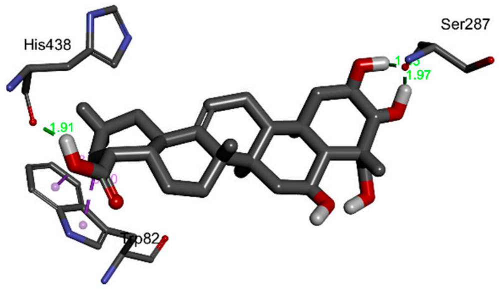

* NB for No Binding affinity; NA for not applicable

Figure S3: ^{1}H NMR and ^{13}C NMR spectra of compounds 1-11.

COMPOUND 1

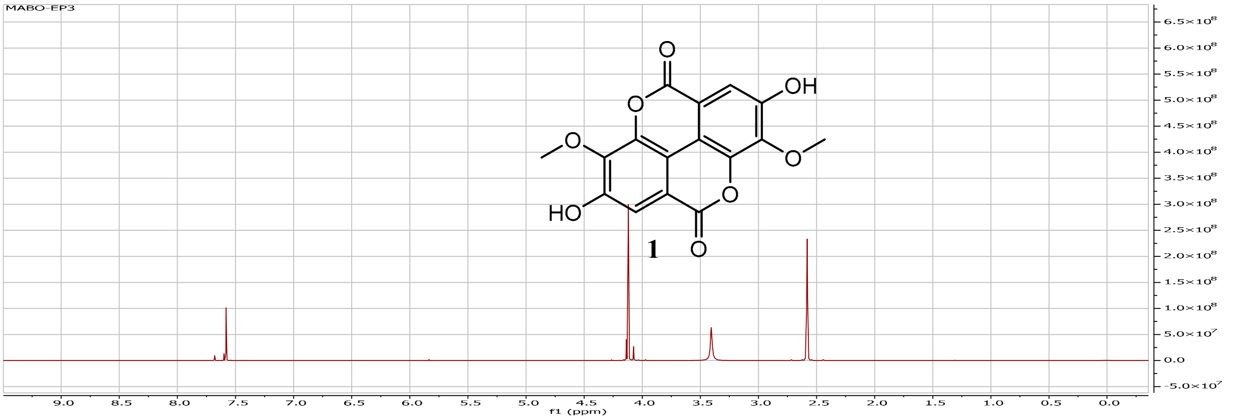

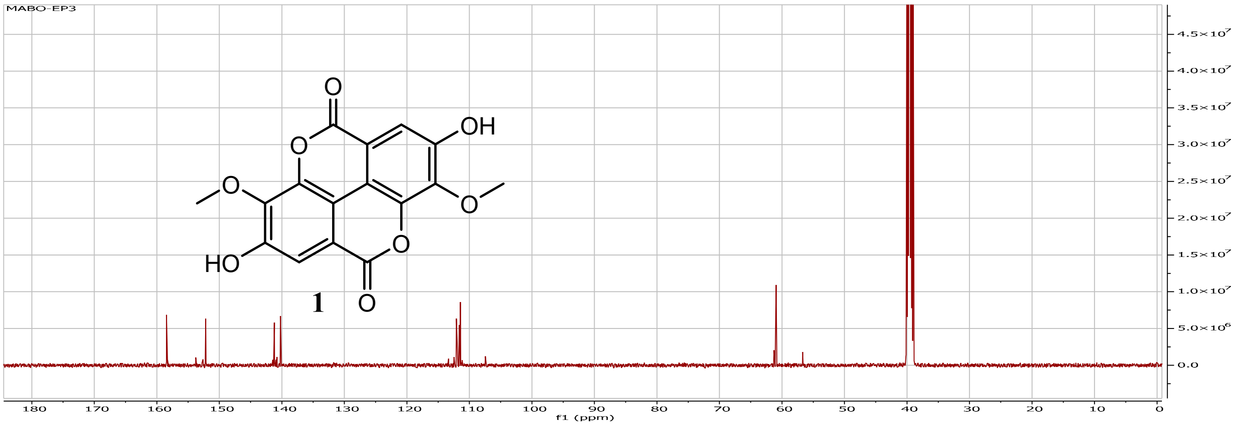

COMPOUND 2

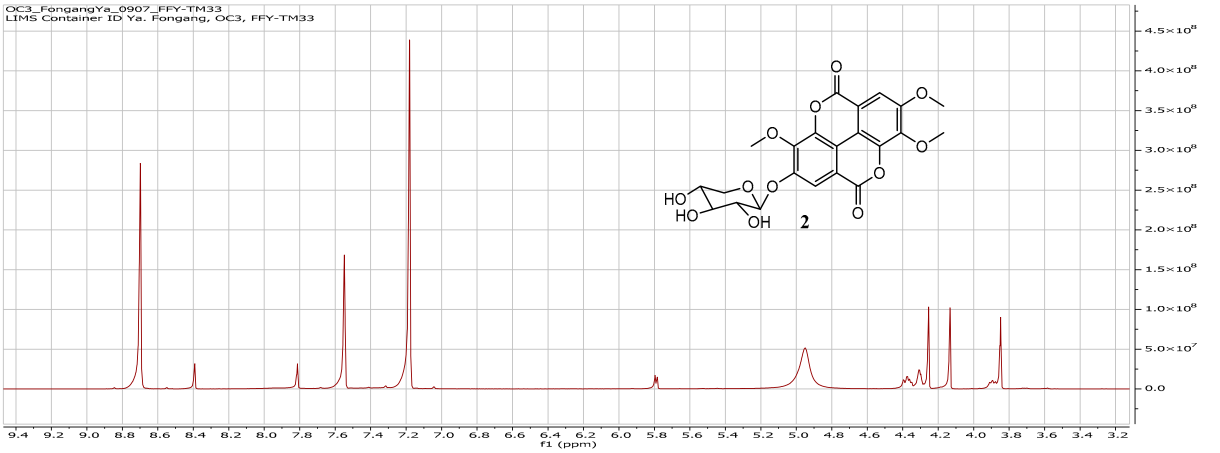

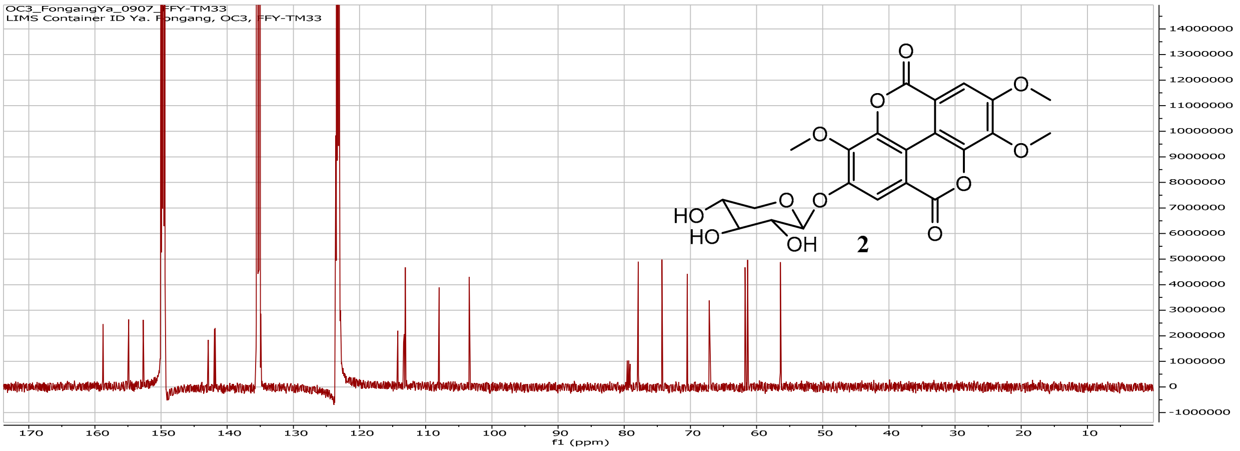

COMPOUND 3

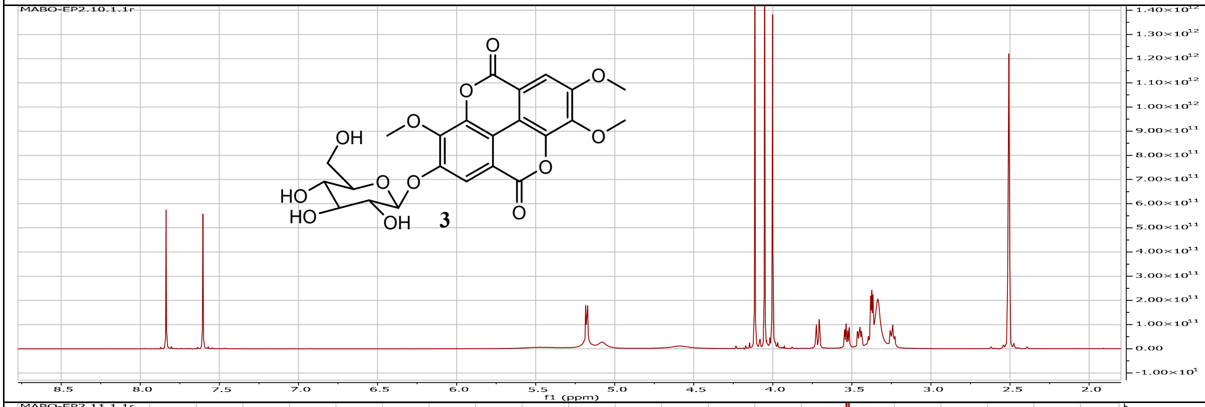

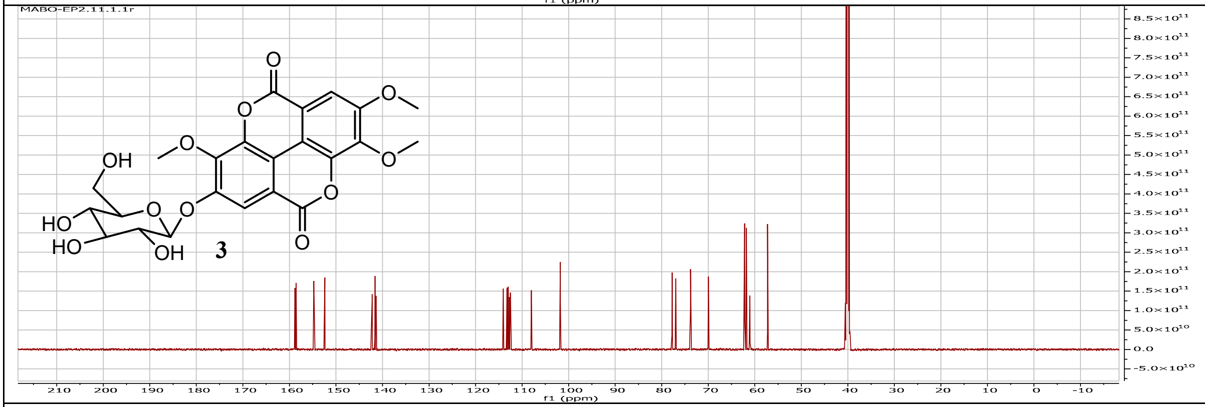

COMPOUND 4

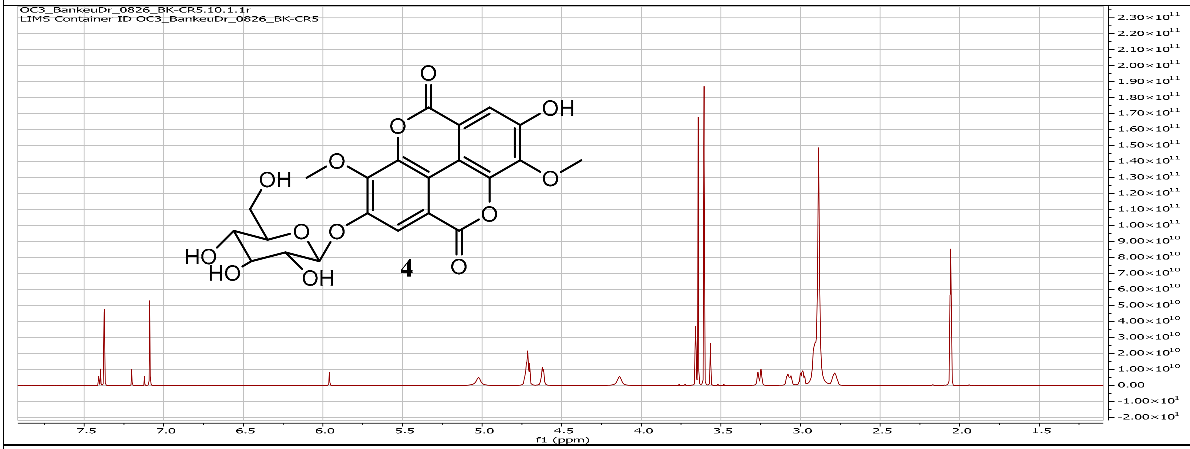

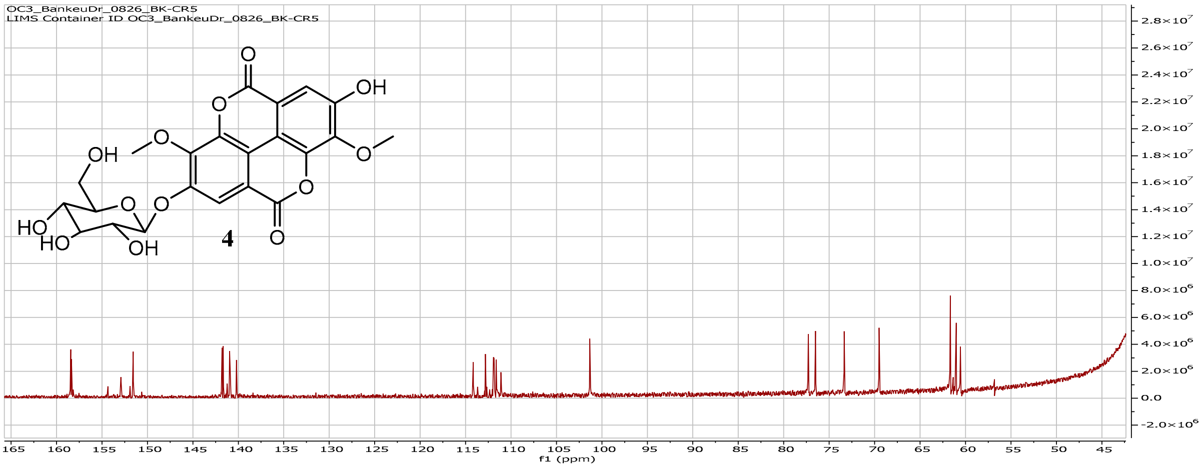

COMPOUND 5

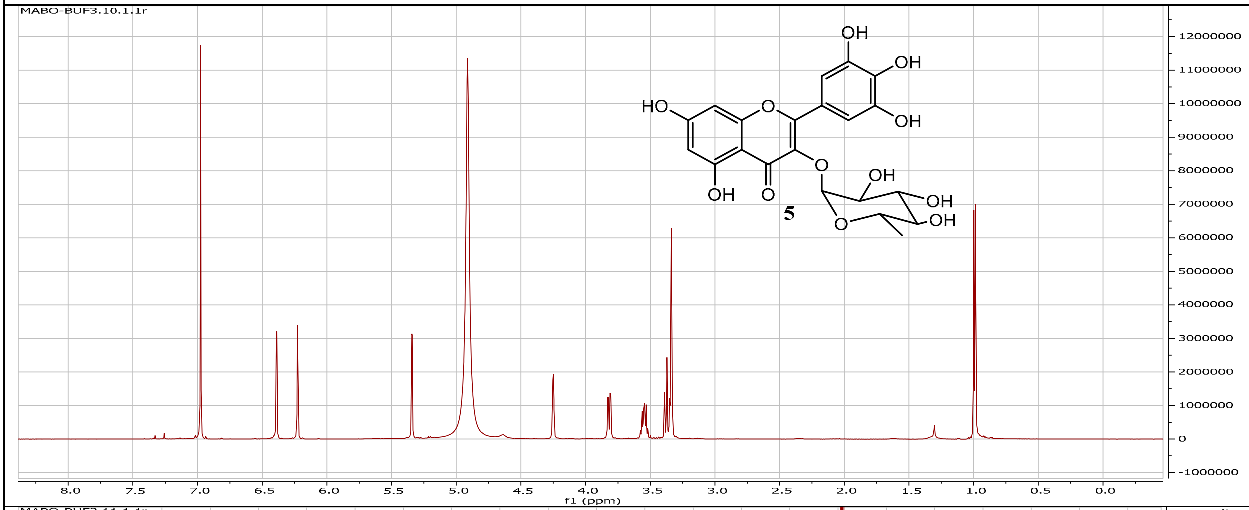

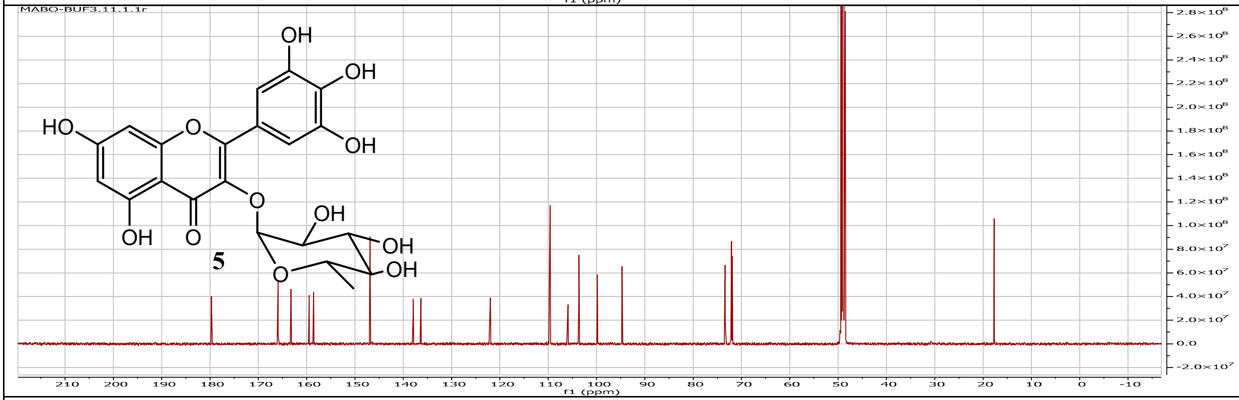

COMPOUND 6

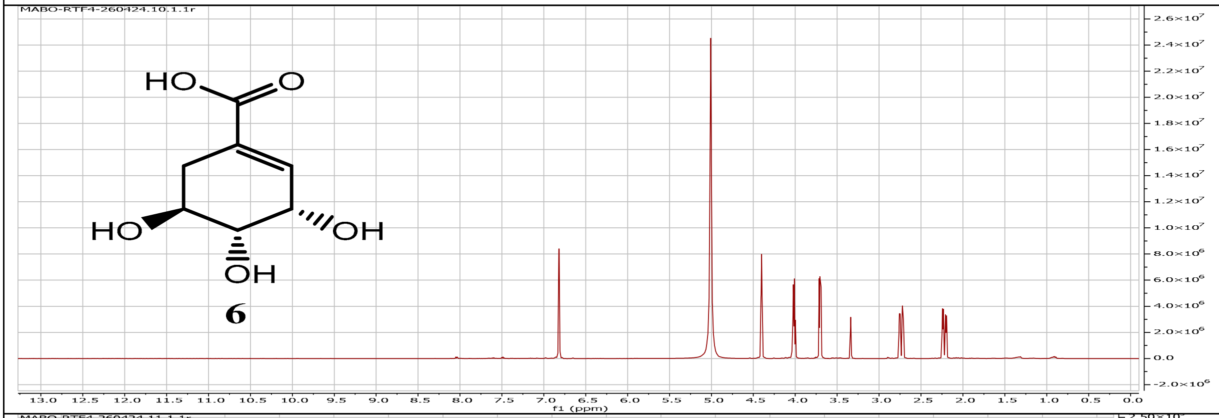

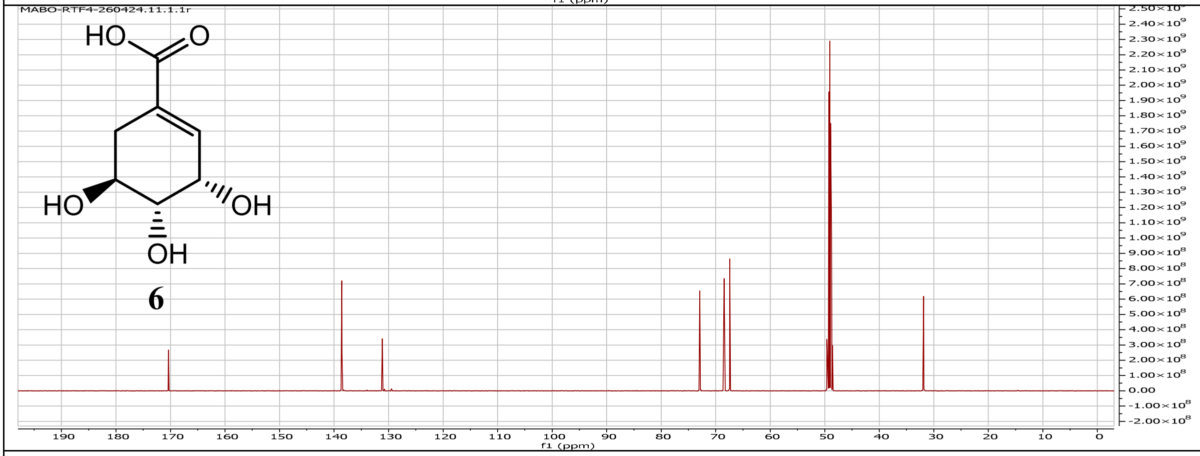

COMPOUND 7

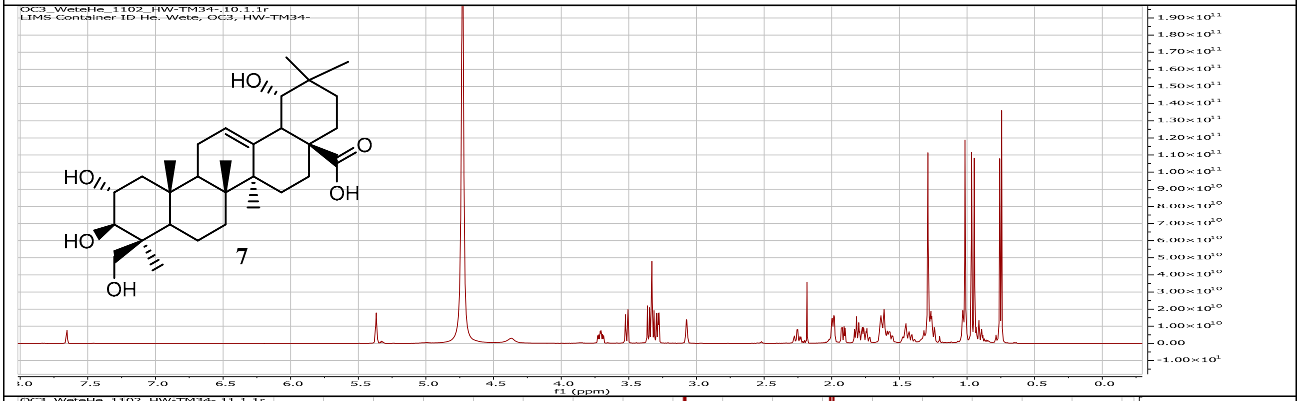

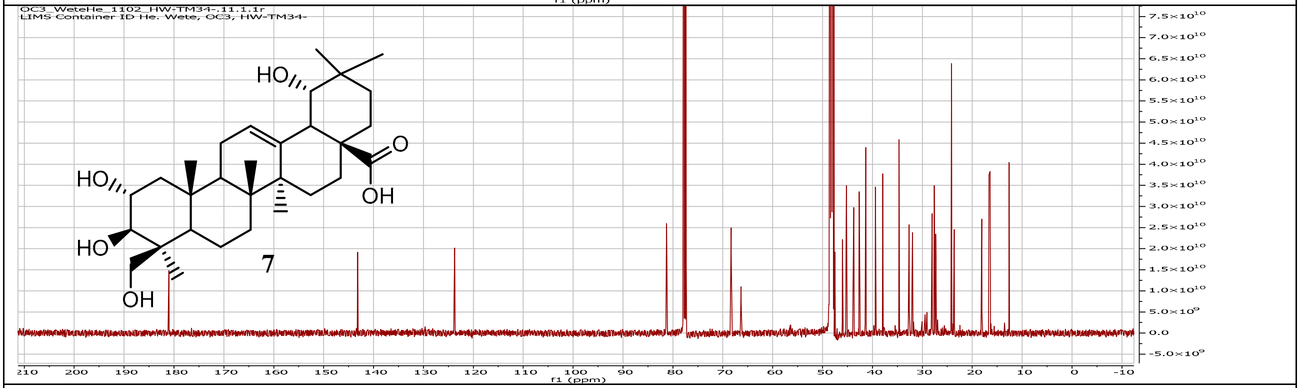

COMPOUND 8

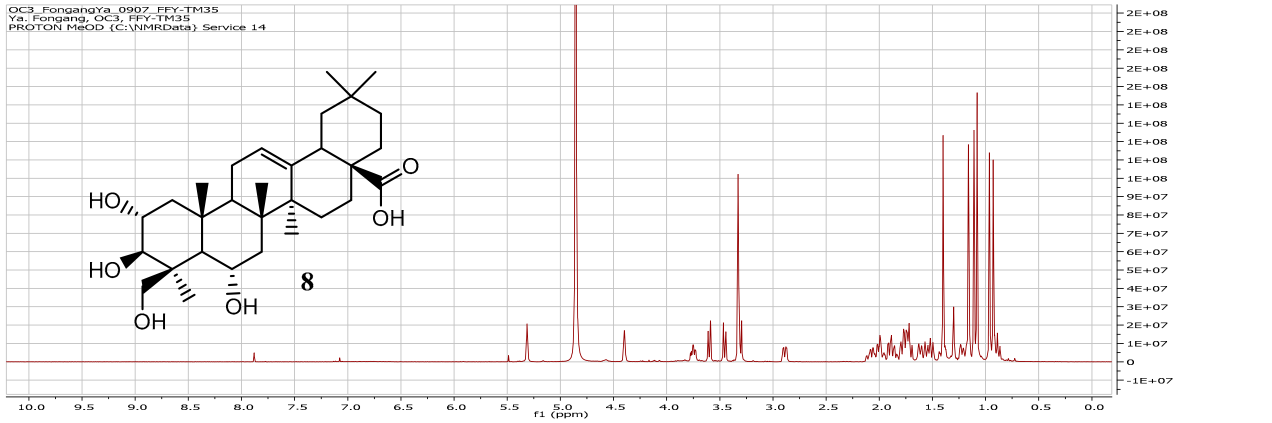

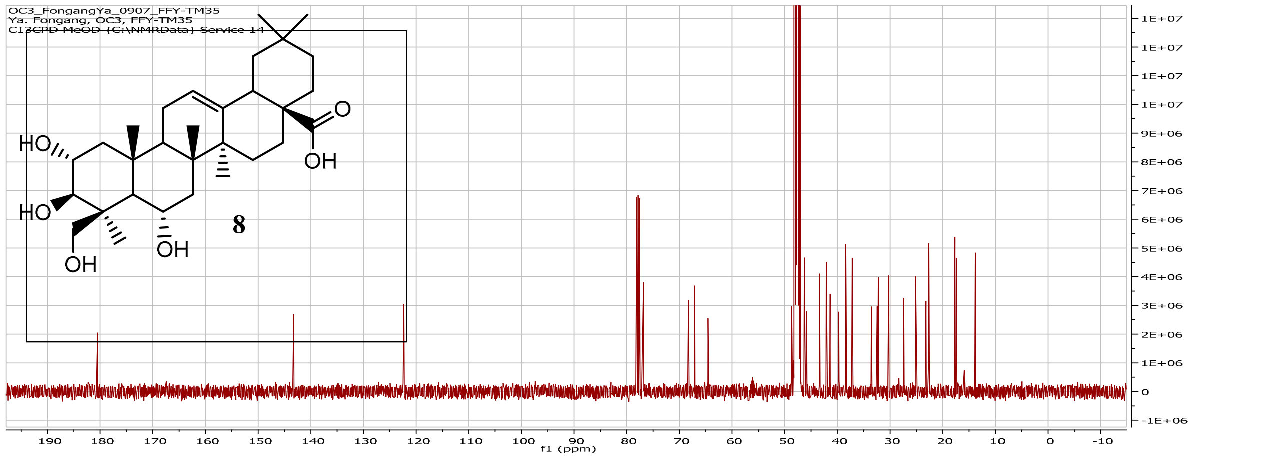

COMPOUND 9

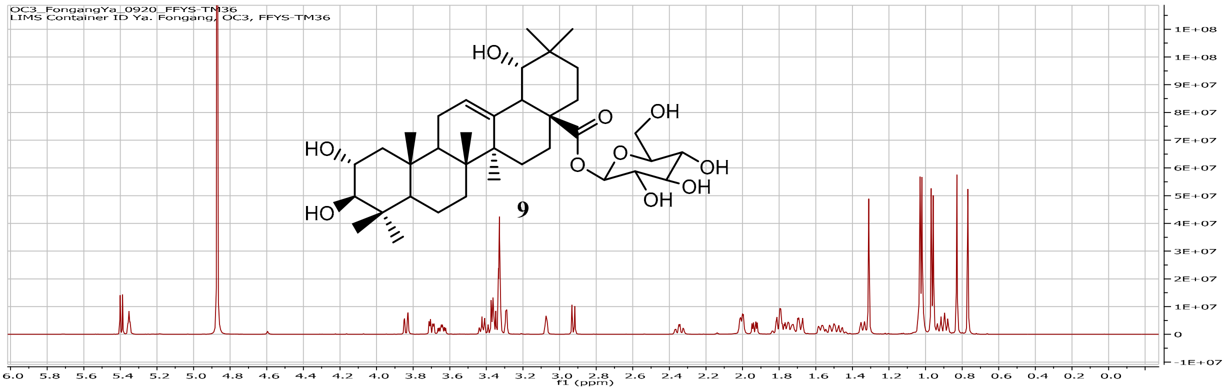

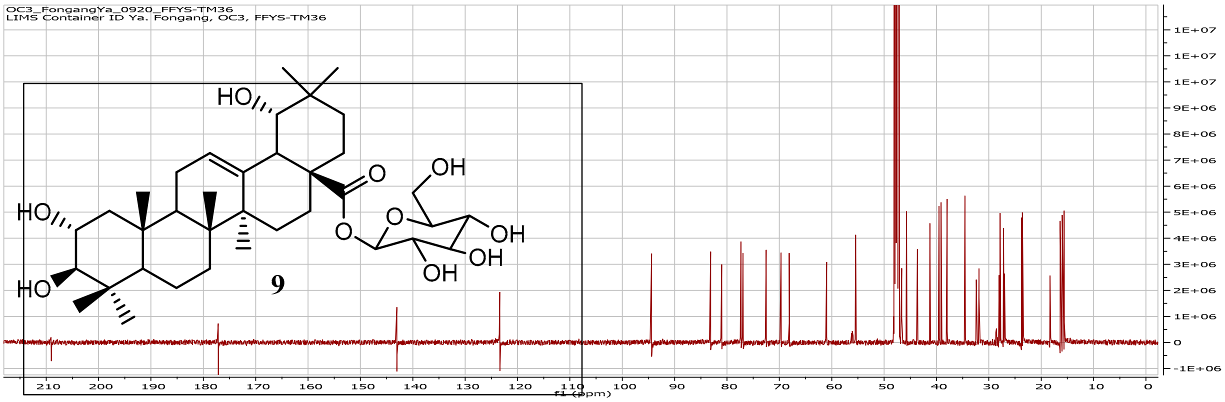

COMPOUND 10

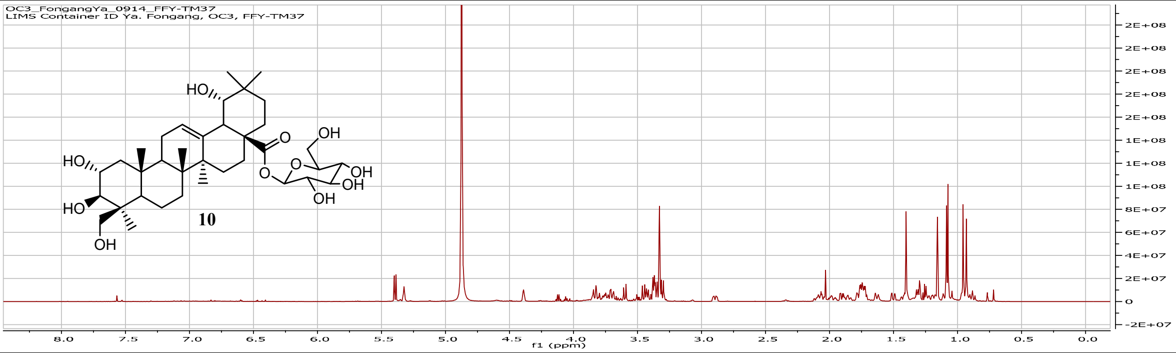

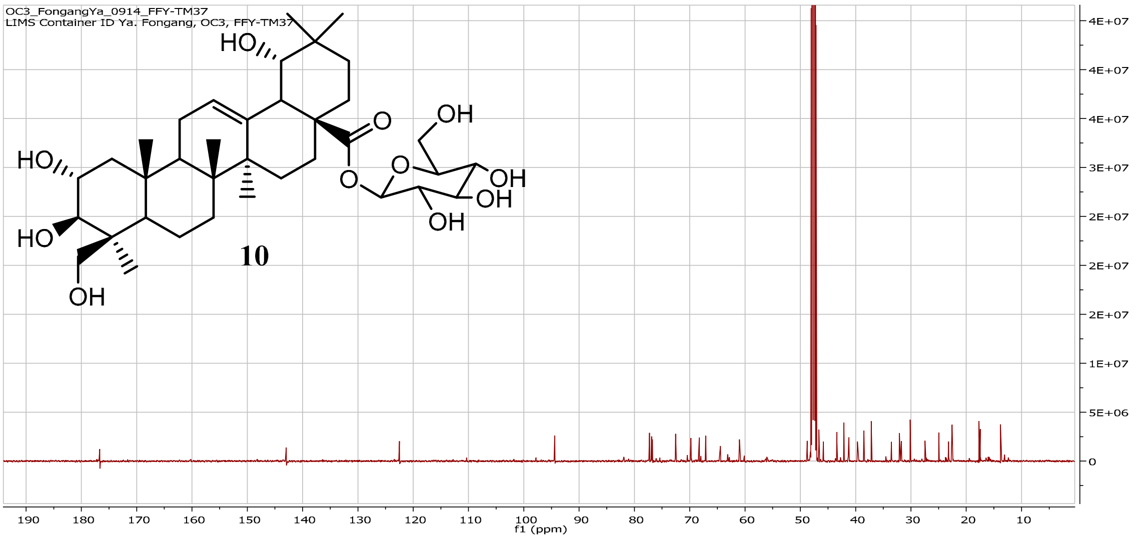

COMPOUND 11

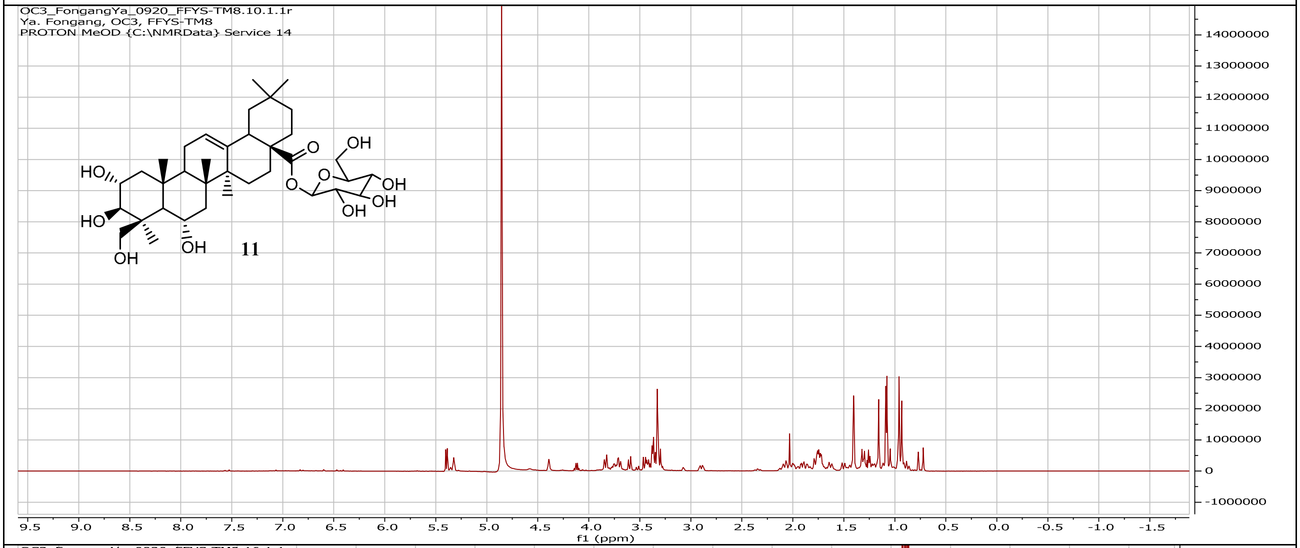
OC3_FongangYa_0920_FFYS-TM8.10.1.1r
Ya. Fongang, OC3, FFYS-TM8
PROTON MeOD {C:\NMRData} Service 14
11
f1 (ppm)

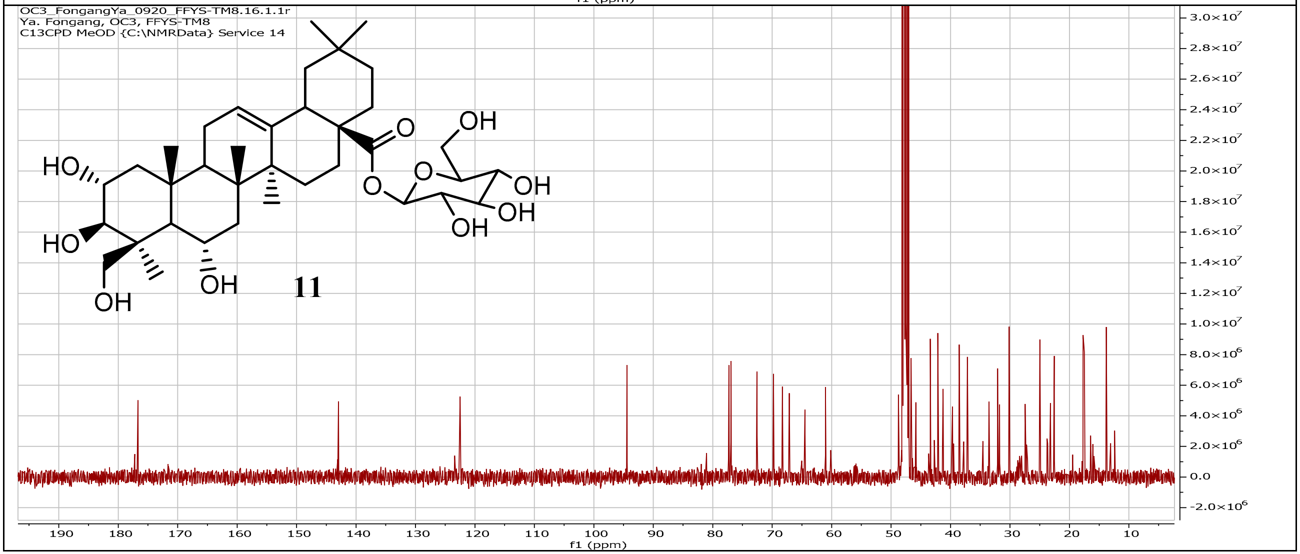
OC3_FongangYa_0920_FFYS-TM8.16.1.1r
Ya. Fongang, OC3, FFYS-TM8
C13CPD MeOD {C:\NMRData} Service 14
11
f1 (ppm)
